# On the Origins of Phenotypic Parallelism in Benthic and Limnetic Stickleback

**DOI:** 10.1093/molbev/msad191

**Published:** 2023-08-31

**Authors:** Laura L Dean, Isabel Santos Magalhaes, Daniele D’Agostino, Paul Hohenlohe, Andrew D C MacColl

**Affiliations:** School of Life Sciences, The University of Nottingham, University Park, Nottingham, UK; School of Life Sciences, The University of Nottingham, University Park, Nottingham, UK; Department of Life Sciences, School of Health and Life Sciences, Whitelands College, University of Roehampton, London, UK; School of Life Sciences, The University of Nottingham, University Park, Nottingham, UK; Water Research Center, New York University Abu Dhabi, Abu Dhabi, United Arab Emirates; Institute for Bioinformatics and Evolutionary Studies, Department of Biological Sciences, University of Idaho, Moscow, ID, USA; School of Life Sciences, The University of Nottingham, University Park, Nottingham, UK

**Keywords:** evolution, genomic, parallel, speciation, ancestry, phylogeny

## Abstract

Rapid evolution of similar phenotypes in similar environments, giving rise to in situ parallel adaptation, is an important hallmark of ecological speciation. However, what appears to be in situ adaptation can also arise by dispersal of divergent lineages from elsewhere. We test whether two contrasting phenotypes repeatedly evolved in parallel, or have a single origin, in an archetypal example of ecological adaptive radiation: benthic–limnetic three-spined stickleback (*Gasterosteus aculeatus*) across species pair and solitary lakes in British Columbia. We identify two genomic clusters across freshwater populations, which differ in benthic–limnetic divergent phenotypic traits and separate benthic from limnetic individuals in species pair lakes. Phylogenetic reconstruction and niche evolution modeling both suggest a single evolutionary origin for each of these clusters. We detected strong phylogenetic signal in benthic–limnetic divergent traits, suggesting that they are ancestrally retained. Accounting for ancestral state retention, we identify local adaptation of body armor due to the presence of an intraguild predator, the sculpin (*Cottus asper*), and environmental effects of lake depth and pH on body size. Taken together, our results imply a predominant role for retention of ancestral characteristics in driving trait distribution, with further selection imposed on some traits by environmental factors.

## Introduction

Parallel occurrence of adaptive phenotypes across similar but geographically separate environments has long fascinated evolutionary biologists. There are two main mechanisms which can explain such a pattern. First, novel adaptive phenotypes may evolve rapidly and repeatedly in response to new ecological opportunity, that is parallel ecological speciation (Nosil 2012). Alternatively, an adaptive phenotype may arise in a single location and disperse into and/or persist only in suitable environments (Wiens 2004; Kozak and Wiens 2006; Hiller et al. 2019). Although these two mechanisms result in the same pattern, they reflect extremely different evolutionary histories: multiple evolutionary origins of the same phenotype versus a single origin. It is therefore necessary to determine which evolutionary history is responsible for apparent parallelism if we are to understand it. There are many definitions for parallel and convergent evolution (Elmer and Meyer 2011; Rosenblum et al. 2014), but here we focus on whether similar phenotypic adaptations share a common ancestral genetic basis.

Parallel ecological speciation may involve multiple de novo mutations, each of which may lead to a similar phenotype but by a slightly different mechanism. In this instance, it is easy to conclude multiple independent origins. However, evolution is not linear but often reticulated, and in many cases, parallel ecological adaptation may involve repeated reuse of standing genetic variation, that is the same, potentially ancient mutation can be introduced to multiple independent populations via admixture (Jones, Grabherr et al. 2012). In this case, parallel populations may be young and have multiple origins, but the mutations responsible for adaptation are shared and may be much older. This scenario is extremely difficult to differentiate from a scenario in which parallel populations themselves have a single origin (Faria et al. 2014; whether that origin resulted from adaptation from standing genetic variation or other possible sources of novel genetic material). However, it is critical that we attempt to distinguish these scenarios in order to understand the underlying processes that shape evolution.

The benthic–limnetic axis of three-spined stickleback in British Columbia (“BC”), Canada, is an archetypal example of ecological divergence and speciation (Schluter and McPhail 1992; Schluter 1996; Hendry 2009; McGee et al. 2013; Arnegard et al. 2014; Magalhaes et al. 2021). It separates bottom-dwelling, benthic individuals, which feed predominantly on macroinvertebrates, from pelagic fish, feeding mostly on zooplankton (McPhail 1992, 1994; Gow et al. 2008). These two freshwater ecotypes are characterized by heritable differences in body size, shape, trophic morphology, and body armor, which confer fitness advantages in their corresponding habitats (Schluter 1995; Hatfield and Schluter 1999; Gow et al. 2007). In BC, stickleback occurs both as sympatric benthic–limnetic species pairs and solitary populations that possess phenotypes along the benthic–limnetic axis (Schluter and McPhail 1992; Rundle and Schluter 1998; Taylor and McPhail 2000; Vines and Schluter 2006; Jones, Chan et al. 2012). Previous work has identified patterns of parallelism in adaptive genomic divergence across benthic–limnetic species pairs but closer genetic affinity within lakes at neutral markers (Taylor and McPhail 1999, 2000; Jones, Chan et al. 2012). This work has tentatively led to the conclusion that benthic and limnetic phenotypes evolved repeatedly and independently in multiple lakes (McPhail 1993; Taylor and McPhail 1999; Jones, Chan et al. 2012). However, gene flow occurs to some extent in all benthic–limnetic species pairs (Gow et al. 2006; Taylor et al. 2006), and even low levels of gene flow quickly erode genetic differences at neutral loci, making it impossible to separate patterns of recent in situ ecological speciation from those derived from secondary contact of much older independent lineages (Bierne et al. 2013). Little investigation has so far been conducted beyond the species pairs, which coexist in only a handful of lakes (McKinnon and Rundle 2002), but see Harer et al. (2021). Populations in solitary lakes have far less opportunity for gene flow and thus will likely give a more reliable estimate of the evolutionary history of benthic and limnetic ecotypes.

We investigate whether benthic–limnetic divergence in BC stickleback likely has a single or multiple evolutionary origins. We first characterize genomic and phenotypic divergence across populations and show that all freshwater individuals fall within one of two genomic clusters, one of which exhibits a more benthic phenotype and the other exhibit a more limnetic phenotype. We construct a maximum likelihood phylogeny using a stringently filtered dataset, removing all known quantitative trait loci (QTLs) in stickleback, and test for phylogenetic signal in ecologically relevant phenotypic traits. We construct a microevolutionary adaptive landscape for the BC radiation using recently available niche modeling techniques (Ingram and Mahler 2013) to identify the best fitting model of benthic–limnetic trait evolution. Finally, accounting for any phylogenetic signal, we test for relationships between phenotype and environment to detect signals of true ecological adaptation.

## Results

**Fig. 1.**
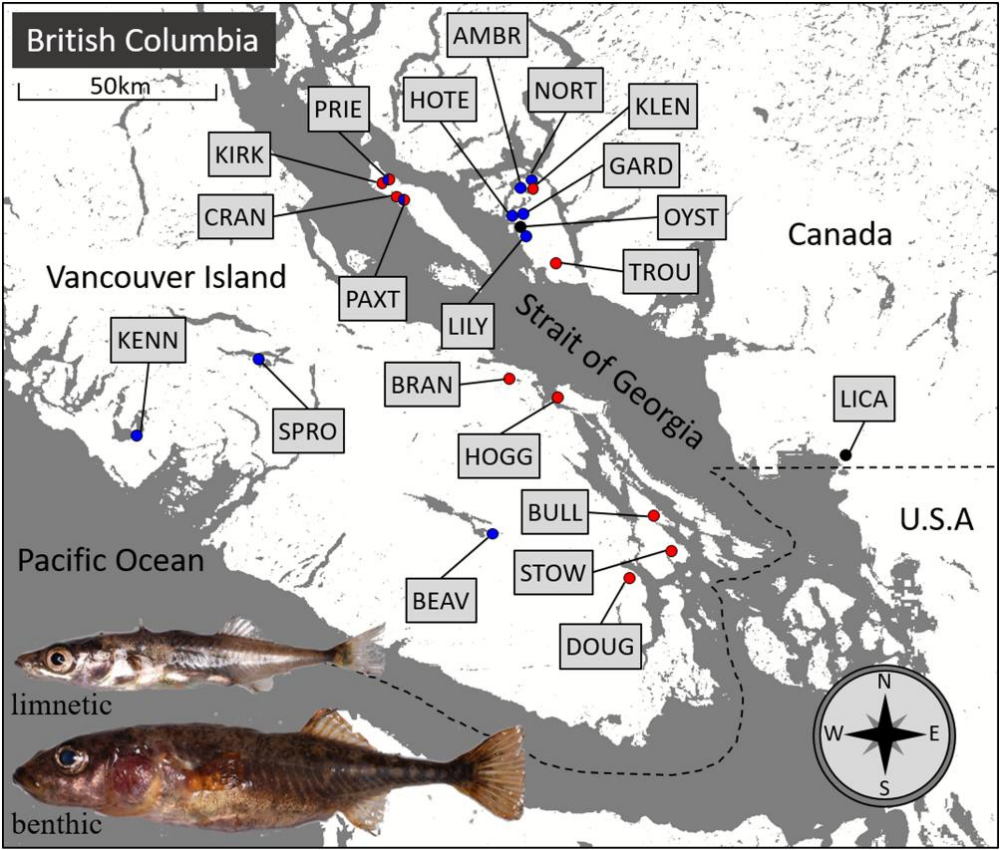
Map of sampling locations in BC, and example photographs of limnetic and benthic stickleback from Paxton Lake. Sample sites are indicated by circles, and their associated lake ID. Black circles indicate marine populations, blue circles indicate populations in cluster 1 of our genomic analyses, and red circles indicate populations in cluster 2. Red and blue semicircles indicate species pair populations containing individuals from both clusters 1 and 2. The dashed line represents the border between Canada and the United States.

We collected stickleback and environmental parameters from 21 lakes surrounding the Strait of Georgia, BC (fig. 1), including two species pair lakes, two coastal locations (representing putative marine ancestors), and 17 solitary freshwater lakes (supplementary table S1, Supplementary Material online). We collected phenotypic data for key benthic–limnetic divergent traits (Materials and Methods) for approximately 32 individuals (mean = 31.5, standard error [SE] = 2.4) and generated RAD-seq genomic data (Magalhaes et al. 2021), for approximately 16 individuals (mean = 15.9, SE = 0.9), from each lake, 333 individuals in total. This resulted in a master genomic dataset of 12,756 single nucleotide polymorphisms (SNPs), which was subject to further filtering for some analyses (table 1).

**Table 1.** SNP Datasets.

Dataset	*N* lakes	*N* individuals	*N* SNPs	LD thinned	Known QTL removed
Dataset 1	21	333	12,756	✗	✗
Dataset 2	21	333	6,215	✓	✓
Dataset 3	5	53	6,215	✓	✓

Note.—*N*, number; LD thinned, SNPs with *r*^2^ > 0.2 removed; known QTL, SNPs within known QTL regions. Details of the SNP datasets used in genomic analyses.

### Genomic Divergence

**Fig. 2.**
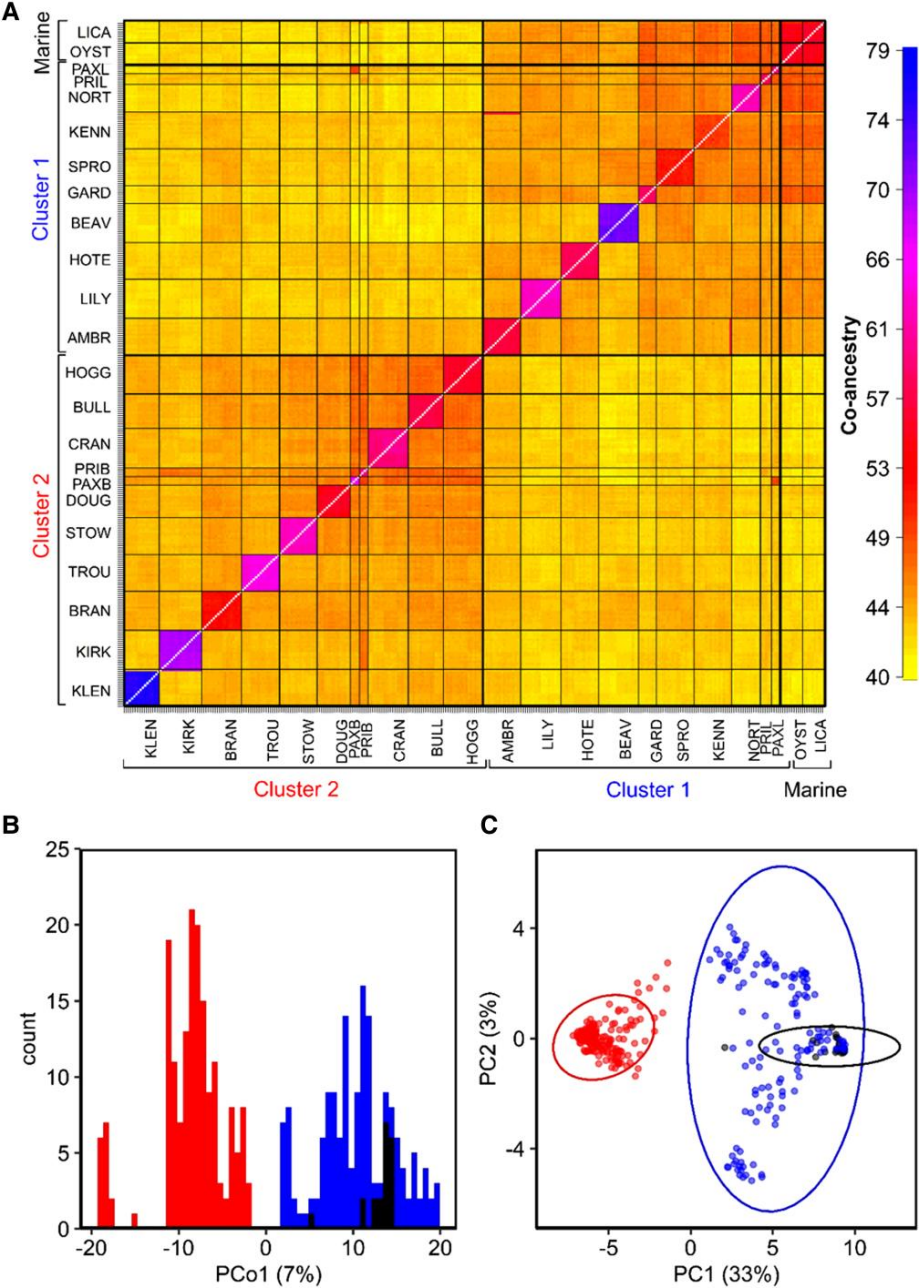
Genetic structure across BC stickleback populations. (*A*) Coancestry matrix of BC stickleback populations. Thin lines separate populations, and thick lines separate the broader genetic clusters. (*B*) Distribution of BC stickleback along the first principal coordinate of a genomic PCoA (dataset 2, 6,215 SNPs). (*C*) Distribution of BC individuals in a PCA of 60 linked SNPs comprising linkage cluster 10, identified by LDna. Circles represent individuals. In (*B*) and (*C*), black represents marine individuals, blue—cluster 1 individuals, and red—cluster 2 individuals.

We used two methods to quantify clustering within the genomic data. First, a coancestry matrix in fineRADstructure (Malinsky et al. 2018) (dataset 1, 12,756 SNPs) identified two genomic clusters across all populations (fig. 2*A*), one incorporating the marine populations and approximately half of the freshwater populations (cluster 1) and the other comprising the rest of the freshwater lakes (cluster 2). Although marine populations formed part of cluster 1, they are considered separately here and in all further analyses because their presence in shallow coastal areas is transient and they likely represent the ancestral phenotypic state of all freshwater populations (Jones, Grabherr et al. 2012). Second, we conducted a principal coordinate analysis (PCoA, dataset 2, 6215 SNPs). The same two broad genomic clusters (1 and 2) were identified by PCoA analysis, separating along PCo1 (7% of total variation, fig. 2*B*).

To further investigate the genomic properties of cluster 1 and 2 differentiation, we conducted a linkage disequilibrium network analysis (LDna) using the LDna R package (dataset 1, 12,756 SNPs). Many evolutionary phenomena (e.g., inversions, selective sweeps, population admixture, genetic drift, and epistatic fitness interactions among loci) result in linkage disequilibrium (LD) among multiple loci. This software is designed to detect independent clusters of linked loci, each of which is a signature of a different evolutionary phenomenon (Kemppainen et al. 2015). Our LD network contained 12 linkage clusters (supplementary figs. S4 and S5, Supplementary Material online). Principal component analysis (PCA) on the SNPs from each cluster revealed a group of 60 SNPs, spread across 17 of the 21 chromosomes in the stickleback genome, associated with cluster 1–cluster 2 separation (fig. 2*C*). Of these 60 SNPs, 28 fell directly within genes (supplementary table S2, Supplementary Material online). Most other LD clusters only separated single populations from all others, likely reflecting local patterns of selection and drift, and none of the LD clusters separated marine and freshwater adapted populations (supplementary figs. S4 and S5, Supplementary Material online).

### Phenotypic Divergence

**Fig. 3.**
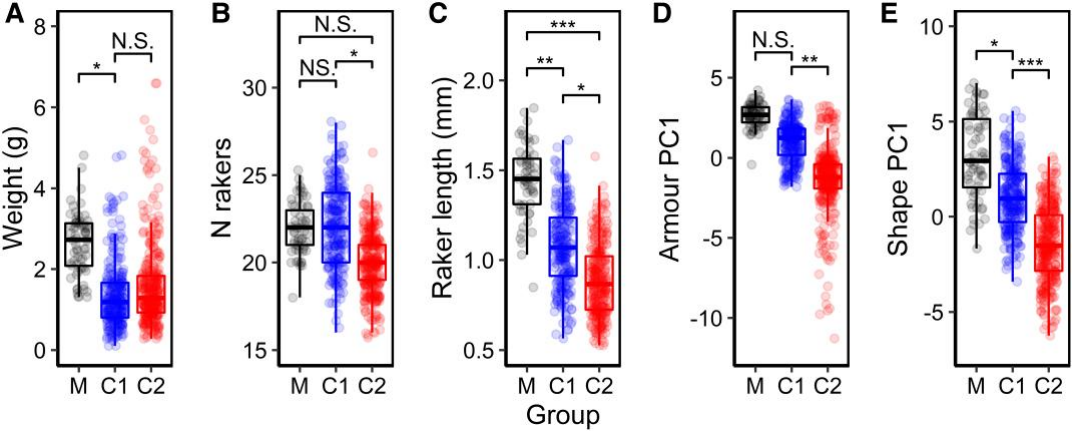
(*A*–*E*) Phenotypic differences between marine fish and two freshwater genetic clusters. Circles represent individuals. Brackets and asterisks indicate significance thresholds of post hoc estimated marginal means tests between groups: NS indicates *P* > 0.05, **P < 0.01, and ***P < 0.001. All *P* values were adjusted for multiple comparisons. M, marine; C1, cluster 1; C2, cluster 2.

To determine whether the genomic clusters differed phenotypically, we analyzed differences in group means for important benthic–limnetic divergent phenotypic traits: weight, gill raker length and number, armor PC1 (increasing size of all armor variables and increasing lateral plate number, explaining 70.4% of body armor variation [Materials and Methods]), and shape PC1 (describing shape changes associated with benthic and limnetic habitats, such as eye size, body depth, and mouth length, explaining 23.2% of body shape variation [Schluter 1993; Willacker et al. 2010]). There were differences in phenotype between the three groups for all phenotypic traits (supplementary table S3, Supplementary Material online). For most traits, clusters 1 and 2 differed from marine fish, and for all traits except for body weight, clusters 1 and 2 differed from each other (supplementary table S4, Supplementary Material online; fig. 3*A–E*). Cluster 1 had a typically limnetic phenotype (Schluter 1993; Willacker et al. 2010) with a smaller size, longer, more numerous gill rakers, more body armor, a larger pelvis relative to spine length, and a more streamlined, slender body shape than cluster 2, which had a much more benthic phenotype (fig. 3*A–E*). Population-level phenotypic data are given in supplementary figure S6, Supplementary Material online.

Coancestry plots of genomic data from Alaskan stickleback populations (gathered and processed in an identical way to BC; see supplementary methods, Supplementary Material online for details) also show two broad genomic clusters, but these represent the geographical distribution of populations (separating the Kenai peninsula from the Matanuska-Susitna [“Mat-Su”] valley) and not benthic and limnetic phenotypic differences (supplementary fig. S1, Supplementary Material online).

### Phylogeny

**Fig. 4.**
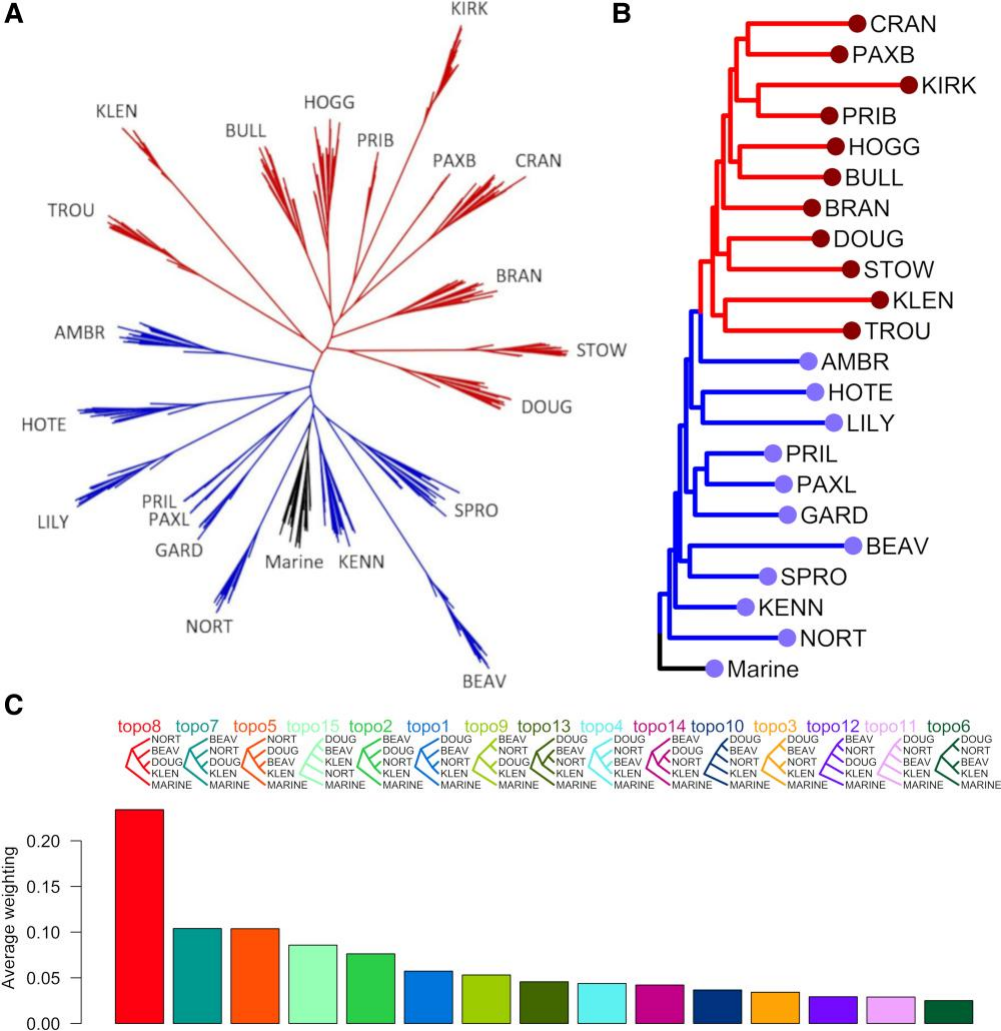
(*A*) Maximum likelihood phylogeny of 333 BC stickleback. Black indicates marine; blue, cluster 1; and red, cluster 2. (*B*) The same phylogeny as (*A*) with monophyletic populations collapsed into single tips. Branch colors in (*B*) denote the same as in (*A*). Colored circles at branch tips represent two independent selection regimes, detected in the optimal model of niche evolution (R package: SURFACE). In both phylogenies, species pairs are divided into benthic (PAXB, PRIB) and limnetic (PAXL, PRIL) populations. (*C*) Mean weightings for all possible topologies for four freshwater populations: two from cluster 1, NORT and BEAV; and two from cluster 2, DOUG and KLEN, with a single individual from the marine population LICA as the outgroup.

Phylogenetic reconstruction for population-level genomic data can be notoriously problematic as numerous factors, including selection and ongoing and/or historic gene flow, can mask true phylogenetic signal in the data (Leache et al. 2014; Som 2015). To minimize bias in our analysis, we first filtered our master dataset (dataset 1, 12,756 SNPs) to remove all SNPs falling within any of the 188,257,608 bp (approximately 41% of the stickleback genome) identified in Peichel and Marques (2017) as containing known QTL in three-spined stickleback. QTLs are loci of large effect and thus most likely to influence tree topologies in phylogenetic reconstruction. They are also extremely well mapped in stickleback (Peichel and Marques 2017), allowing us to avoid potential biases caused by selection on any of these loci. QTLs that were removed included those responsible for benthic–limnetic differences in body shape, defense (antipredator armor), feeding (trophic morphology), and pigmentation. Removing all known QTLs left 8351 SNPs. We then filtered for linkage disequilibrium (*R*^2^ > 0.2, leaving 6215 SNPs, dataset 2). We constructed a maximum likelihood phylogeny for all populations using RAxML. Consistent with the coancestry and PCoA analyses, the topology showed clusters 1 (more limnetic phenotype) and 2 (more benthic phenotype) at opposite ends of the tree, with marine fish most closely related to cluster 1 (fig. 4*A*). The two species pair lakes both contained limnetic individuals whose closest relatives were in cluster 1 (PAXL and PRIL) and benthic individuals whose closest relatives were in cluster 2 (PAXB and PRIB, fig. 4*A*). Alternative approaches to filter our master SNP set, for example removing all SNPs with Fst between clusters ≥0.35 (mean Fst: 0.08) or Fst between lakes with sculpin present (a major selective agent for stickleback) or absent ≥0.35 (mean Fst: 0.07), before phylogenetic reconstruction with RAxML made no difference to the distinction of clusters 1 and 2 in either topology (supplementary fig. S2 and supplementary methods, Supplementary Material online).

Sticklebacks that inhabit streams typically exhibit a more benthic phenotype than lacustrine populations (Stuart et al. 2017), but current research suggests that most of the lake–stream populations on Vancouver Island evolved independently in each watershed and are each other's closest relatives (Stuart et al. 2017). Therefore, as an additional test of whether selection for a more benthic phenotype in stream habitats overwhelms the phylogenetic signal in genomic data, we performed a supplementary reanalysis of a subset of the RADseq genomic data for lake–stream population pairs on Vancouver Island, collected by Stuart et al. (2017). We selected 6 lake–stream pairs (201 total individuals) from across Vancouver Island for reanalysis (supplementary fig. S3*A*, Supplementary Material online). We processed the data in an identical manner (so far as possible) and applied identical filtering as for the dataset used for phylogenetic construction in our analysis (fig. 4*A*, as a further test for the adequacy of our SNP filtering approach). Lake and stream stickleback were monophyletic by watershed in all six cases (supplementary fig. S3*B*, Supplementary Material online), suggesting that stream stickleback likely did evolve independently in each watershed and selection for a more benthic phenotype in streams was not sufficient to affect the phylogeny. We also performed a supplementary phenotypic analysis showing that stream fish from Vancouver Island are quantifiably phenotypically different from the benthic lake fish in our analysis (supplementary fig. S3*C*, Supplementary Material online).

We also performed a topology weighting analysis on a subset of populations selected specifically to test the likelihood that the phenotypes associated with clusters 1 and 2 could have evolved repeatedly in situ. Topology weighting is a means by which to quantify relationships between taxa that are not necessarily monophyletic. It determines how support for each possible topology varies across the genome and allows quantification of the overall proportion of the genome which supports each possible tree. This allowed us to identify multiple highly supported phylogenies, so that we could determine whether any of those with high support involved a model in which the two clusters arose more than once. It also allowed us to quantify what proportion of the genome supports our most likely topology and how big the difference is between this and the level of support for the next most likely tree. To do this, we selected pairs of populations that were from different cluster, but were geographically as close together as possible. We did this twice with the two pairs being geographically as far apart as possible (DOUG and BEAV, and KLEN and NORT, approximately 100 km and on opposite sides of the Georgia Strait). We did this so that if much of the genome supported a “geographic” model, in which populations near to each other were more closely related, we would be as likely as possible to detect it. One marine individual was used as the outgroup. The topology with the highest weighting across all 50 bp sliding windows (topology 8) was concordant with the maximum likelihood phylogeny, with the two cluster 2 populations (DOUG and KLEN) forming a monophyletic clade and each cluster 1 population splitting off earlier, deeper to the root (fig. 4*C*). The topology with the second highest weightings (topology 7) was also concordant and just involved a switching of the order in which the cluster 1 populations split from the root. The simple geographical hypothesis, with the two pairs of populations nearest to one another being most closely related (topology 3), received very little support. The highest ranking topology had more than twice the proportional support that the second most likely topology had, suggesting that there is a strong genome-wide signal in favor of the maximum likelihood topology.

### Phylogenetic Signal

If benthic and limnetic phenotypes had resulted from repeated, rapid adaptive divergence, phylogenetic signal (the tendency for more closely related individuals to share phenotypes) would be obscured, and trait distributions would instead mimic the adaptive landscape—that is variation in the relevant environmental characteristics. Therefore, we tested a null model that traits would be distributed randomly with respect to phylogeny, and an association of trait distribution with population-level relatedness was taken as evidence that benthic and limnetic niches were conserved from the ancestral state (Buckley et al. 2010; Wiens et al. 2010; Muenkemueller et al. 2012).

We estimated phylogenetic signal at the population level, using mean phenotypic trait data, and collapsing nodes in the phylogeny by population (with the two marine populations grouped into a single node, as they lacked monophyly), using the R package PhyloSignal. We also tested five simulated traits that had no true association with phylogeny. We identified phylogenetic signal in all five real phenotypic traits: weight, gill raker number, gill raker length, armor PC1, and shape PC1 (*P* values <0.05, table 2). None of the five randomly simulated traits showed phylogenetic signal (*P* values >0.05, table 2).

**Table 2.** Phylogenetic Signal in Real and Simulated Phenotypic Traits.

Trait	Pagel's λ	*P* value
Weight	1.5881	**0**.**0421**
Number of gill rakers	2.1535	**0**.**0010**
Mean gill raker length	1.6492	**0**.**0032**
Armor PC1	0.9667	**0**.**0269**
Shape PC1	1.3647	**0**.**0010**
Random 1	0.0001	1.0000
Random 2	0.0001	1.0000
Random 3	0.0001	1.0000
Random 4	0.0001	1.0000
Random 5	0.0001	1.0000

Note.—The table shows estimates of phylogenetic signal (Pagel's λ) and their associated *P* values. *P* values <0.05 are highlighted in bold.

### Niche Evolution Modeling

Niche evolution modeling combines phylogenetic information with the distribution of phenotypic traits across the tree to identify the most likely number and location of selection regimes imposed across the whole phylogeny. It also identifies the number of instances of convergence (where the same regime appears multiple times across the tree). If the benthic phenotype had evolved repeatedly and independently across the phylogeny, niche evolution modeling should identify multiple instances of convergence of a benthic selection regime. We performed niche evolution modesling using the R package SURFACE (Ingram and Mahler 2013). We ran SURFACE using the same collapsed phylogeny and associated trait data that were used to estimate phylogenetic signal. The best fitting model involved two different selection regimes across the phylogeny (fig. 4*B*). The first included all marine and cluster 1 populations, and the second, all cluster 2 populations. The best fitting model included no instances of convergence between selection regimes, that is each independent regime appeared only once across the phylogeny.

### Relationship between Phenotype and Environment

To test for phenotype–environment relationships, we used linear mixed models, following a phylogenetic generalized least squares (PGLS) approach so that phylogenetic signal could be accounted for and fitted to the population means of phenotypic traits in R. Marine fish were excluded from all phenotype–environment modeling, because of the difficulty of measuring the environment of migratory marine fish. We found that freshwater fish had more body armor in the presence of sculpin (adjusted *P* value <0.05; supplementary table S5, Supplementary Material online), and fish were heavier in deeper lakes and lakes with a higher pH (adjusted *P* values <0.01 and <0.05, respectively). Lake surface area and calcium concentration did not affect any aspect of phenotype, and none of the environmental variables we measured affected the number of gill rakers, the length of gill rakers, or shape PC1 (supplementary table S5, Supplementary Material online).

## Discussion

The repeated occurrence of similar phenotypes in geographically isolated, but similar environments have several possible evolutionary explanations. Perhaps, this pattern results from parallel ecological speciation, or maybe similar phenotypes have a single origin and have subsequently become widely dispersed into suitable habitats. It is impossible to separate these different models using only phenotypic data or small numbers of genetic markers and remains difficult even with genomic data (Faria et al. 2014). Nevertheless, it is important that we attempt to do so, because of the consequences for our understanding of evolution. Parallel evolution has been implicated in the adaptation of stickleback to freshwater (Jones, Chan et al. 2012), but in-depth analyses of global populations suggest that parallel reuse of standing genetic variation has played much more of a role in the older Eastern Pacific populations than in the rest of the species range (Fang et al. 2020). Outside of the Eastern Pacific, many of the freshwater adaptive alleles were likely lost from the pool of standing genetic variation upon colonization of the Atlantic basin and thus were not available for parallel reuse (Fang et al. 2020). Furthermore, there are a number of other cases in which conclusions of parallel speciation have been called into question by the confounding possibility of a single evolutionary origin followed by migration/dispersal and gene flow (Bierne et al. 2013).

We investigated the evolutionary origins of divergent phenotypes in a classic model system for adaptive radiation and ecological speciation. We find strong evidence for a monophyletic clade of stickleback with a benthic phenotype, distributed across freshwater lakes in the southern Georgia Strait region of BC. The evidence strongly suggests that this clade has a single evolutionary origin and is derived from a local ancestor with a more limnetic phenotype. The benthic clade also encompasses benthic fish from two benthic–limnetic species pairs. Our results are therefore consistent with a single origin for lacustrine benthic fish in BC. This contradicts the currently favored model for the evolution of benthic and limnetic stickleback, involving repeated independent evolution of the benthic phenotype in multiple lakes (Taylor and McPhail 1999, 2000; Jones, Chan et al. 2012), highlighting the challenging nature of phylogenetic reconstruction at the population level. Nevertheless, our interpretation is consistent with recent work showing: 1) a split between benthic and limnetic fish in species pairs lakes that likely predates the formation of the lakes (Wang 2018), 2) that crosses between benthic populations exhibit less hybrid breakdown than expected (Thompson and Schluter 2022), 3) more sharing of QTL than expected (Conte et al. 2015; Poore et al. 2023), and 4) patterns of linkage disequilibrium in Pacific three-spined stickleback, which suggest an older evolution of a freshwater ecotype (Fang et al. 2020).

Many factors, such as incomplete lineage sorting, hybridization, gene duplication, natural selection, and recombination, can lead to genealogical discordance in estimations of phylogenetic relationships (Degnan and Rosenberg 2009). Resolving the true relationships between divergent groups can therefore be challenging and require a large number of genetic markers. Much of the published research on benthic and limnetic stickleback in BC has been based only on mitochondrial haplotypes (Taylor and McPhail 1999) or relatively small SNP sets (<1,000 markers) (Jones, Chan et al. 2012) (with some exceptions, discussed below) and has focused heavily on the species pairs. In species pairs, multiple QTL regions are repeatedly responsible for benthic adaptation, which is consistent with a single benthic origin, but neutral SNPs imply closer genetic affinity of benthics and limnetics within lakes (Jones, Chan et al. 2012), consistent with multiple independent origins. However, elevated levels of genetic similarity at neutral markers in species pairs would be expected with even low levels of gene flow and thus may not reflect true phylogenetic relationships (Taylor et al. 2006). Clearly, it is important also to consider relationships in solitary populations, where opportunity for gene flow is greatly reduced.

Harer et al. (2021) looked both at species pairs and solitary populations and identified genomic parallelism in the former but not the latter. However, it is possible that not all genotype–phenotype associations were identified because only an indirect measure of phenotype (lake surface area) was used for genomic correlation (Harer et al. 2021). Miller et al. (2019) also constructed a haplotype network including some of the solitary populations studied here, based on mitochondrial control region sequence containing 25 SNPs. They did not identify monophyly of populations from our benthic clade. However, introgression of mitochondrial DNA can lead to mitochondrial phylogenies that do not reflect the true population history (Pedraza-Marron et al. 2019), and this phenomenon is known to occur in stickleback (Yamada et al. 2001; Kakioka et al. 2020). The haplotypes in Miller et al. (2019) are likely considerably older than the divergence between benthic and limnetic stickleback in BC (five mutational steps occur between the two main haplotype groups in [Miller et al. 2019], which is only one step less than that which separates Atlantic mitochondrial haplotypes that are estimated to be ∼128,000–171,000 years diverged [Makinen and Merila 2008]). With incomplete lineage sorting and/or old gene flow, both of which are highly likely in stickleback (Orti et al. 1994; Taylor and McPhail 1999; Lescak, Marcotte et al. 2015), these mitochondrial haplotypes may well not agree with nuclear genomic patterns. Thus, small chunks of mitochondrial DNA sequence alone may not be enough to establish recent historical events (Hurst and Jiggins 2005). Wang (2018) present a high-resolution nuclear phylogeny (based on >9 million genome-wide SNPs) of four benthic–limnetic species pairs, two of which are included in the present study. This phylogeny, with the exception of Enos Lake, which contains a collapsed species pair (Taylor et al. 2006), shows monophyly of all benthics and all limnetics. This is consistent with our results and suggests a single evolutionary origin for each of these forms.

Some argument remains among evolutionary biologists about whether pervasive, genome-wide selection can overwhelm the signal from other markers and obscure tree topologies (Degnan and Rosenberg 2009; Edwards 2009). Sculpin (*Cottus asper*) are an intraguild predator of stickleback, which, when present, select for a more limnetic phenotype (Miller et al. 2015; Miller et al. 2019). Our results were consistent with this as we identified an effect of sculpin presence on body armor after accounting for phylogenetic signal in our analysis. However, we find the possibility that selection from sculpin obscures the true relationships between populations in our phylogeny unlikely for a number of reasons. First, such a phenomenon is certainly possible in studies using only a small number of markers (Castoe et al. 2009), but with many thousands of unlinked genetic markers, such as in this case, the probability of selection overwhelming the signal from neutral markers is very low (Edwards 2009; Edwards et al. 2016). Second, to reduce the possible effects of selection, we removed all SNPs in our dataset that fell within known QTL prior to phylogenetic reconstruction. QTLs are extremely well mapped in stickleback, and thus, this entailed discounting SNPs from ∼ 41% of the genome (Peichel and Marques 2017). Many of the QTLs that were removed were also specifically for traits known to differentiate benthic and limnetic stickleback in BC and/or identified using crosses from the same populations sampled in this study. Therefore, most potential effects of selection were removed before the phylogeny was constructed. Third, recent modeling suggests that even with strong selection affecting 10–20% of markers, in most instances, phylogenetic inference remains robust to the effects of selection (Adams et al. 2018). Selection from sculpin likely affects less than 2% of the genome (Miller et al. 2019) and thus is at least an order of magnitude smaller in effect size than would be necessary to obscure the true tree topology in this case. Fourth, sculpin were present in some lakes containing the benthic clade and not in all lakes containing the limnetic clade. Thus, the genomic groups identified here do not simply mirror the occurrence of this selective agent but rather represent a deeper set of ancestral relationships.

Stream fish tend to possess a more benthic phenotype than lake fish across the global distribution of stickleback (Berner et al. 2008; Deagle et al. 2012; Stuart et al. 2017). This differentiation is also apparent in BC (Berner et al. 2008; Stuart et al. 2017), and so we also investigated the evolutionary history of stream fish across Vancouver Island, sampled by Stuart et al. (2017). We showed that stream fish in BC are quantifiably phenotypically different from the lacustrine benthic set of populations we identified and that with the exact same data processing and filtering approach, stream fish are phylogenetically most closely related to lake fish within the same catchment and therefore likely did evolve repeatedly and not as a result of the further spread of the benthic clade identified in this manuscript. This additional analysis constituted a further test of our SNP filtering approach, because if selection for a more benthic phenotype in the stream environment was sufficiently strong to overwhelm true phylogenetic signal in the filtered genomic data, we would expect to see monophyly of all stream populations in the phylogeny, but in fact we see the opposite (clustering by watershed), and consequently, any remaining effects of selection in the data were not sufficient to obscure the tree topology.

If the benthic phenotype in lacustrine populations in BC has a single origin, a clear question that requires explanation is how this ecotype spread across BC. Although the lakes containing stickleback in BC are not particularly widespread, they are physically separated by land or sea, which likely makes dispersal a challenge for freshwater stickleback. We speculate that evidence for a large flood (∼500 km^3^ of water) in the Fraser River valley, dated approximately to the end of the Pleistocene and caused by the failure of a large ice dam (Clague et al. 2021), could provide an explanation. The estimated extent of the flood across the southern Georgia Strait is very similar to the current known distribution of benthic stickleback in BC, raising the tantalizing possibility that it may have been responsible for the spread of the benthic lineage of stickleback from a palaeolake in the Fraser Valley, consistent with previous inference about the evolution of Eastern Pacific freshwater stickleback (Fang et al. 2020).

Lacustrine benthic–limnetic species pairs only occur in a handful of small, low elevation lakes in five catchments around the Georgia Strait, all within ∼60 km of one another (Rundle and Schluter 2004). McPhail (1994) pointed out that this extremely restricted geographical distribution must be addressed in any model attempting to explain their evolutionary origin. Since the evidence does not support fully sympatric divergence (Schluter and McPhail 1992; McPhail 1993), he originally proposed a “double-invasion” scenario, in which changes in sea-level facilitated two colonization events of these lakes from a homogenous marine population (McPhail 1993). Modeling of historical changes in sea-level for the region does not provide much support for the double-peak that would have been necessary for this (Josenhans et al. 1997; James et al. 2009; Fedje et al. 2018), but the aforementioned flood could have also provided a potential mechanism. Regardless of the means by which the colonizations occurred, we propose that the double colonization of these lakes may have been made not by two influxes of a homogenous marine population but by populations who had already experienced a period of evolution in allopatry and thus were to some degree differentiated. This alteration to McPhail's original hypothesis would fit with evidence that the split between benthic and limnetic fish in species pairs lakes likely predates the formation of the lakes (Wang 2018), that crosses between benthic populations exhibit less hybrid breakdown than expected (Thompson and Schluter 2022), and that there is more sharing of QTL than expected (Conte et al. 2015; Poore et al. 2023).

Our investigations have shown that the well-studied benthic–limnetic species pairs should be understood as part of a broader radiation along the benthic–limnetic axis in BC. We highlight the need to consider carefully all possible explanations for the occurrence of parallel phenotypes if we are to achieve a proper understanding of the evolutionary processes that mediate divergence. Sticklebacks are clearly capable of remarkably rapid ecological adaptation (Kitano et al. 2008; Aguirre and Bell 2012; Terekhanova et al. 2014; Lescak, Bassham et al. 2015; Roberts Kingman et al. 2021), but we have shown that the retention of ancestral characteristics can also be important in explaining the distribution of divergent phenotypes. This has significant implications for how we think about the process of evolution and raises the possibility that other model examples of in situ ecological adaptation may also result from dispersal rather than convergence.

## Materials and Methods

### Samples Sites and Environmental Measurements

A total of 21 lakes surrounding the Strait of Georgia, BC, which were likely to vary substantially in the ecological niches they presented to stickleback (because of variation in environmental factors), were selected for sampling (see supplementary table S1, Supplementary Material online for detailed sample site information and fig. 1 for a map of sampling locations). This included two lakes, Paxton (PAXT) and Priest (PRIE), known to contain benthic–limnetic stickleback species pairs (McPhail 1992, 1993), and two coastal locations accessible from the sea, Oyster lagoon (OYST) and Little Campbell River (LICA), where marine fish are present during the spring breeding season.

The size and depth of a lake largely determine whether both benthic and limnetic habitats are present (in larger deeper lakes) or just benthic (in small, shallow lakes). Therefore, we measured the surface area (km^2^) using GoogleEarth and collected data on the mean depth (m) of each lake from either HabitatWizard (accessed January 27, 2020) or from data collected in Vamosi (2003), with permission. The presence of other fish species can also determine whether both, one, or none of those niches are available to stickleback (Vamosi 2003). Many other fish species occur in BC, some of which are predators and/or competitors of stickleback. Cutthroat trout (*Oncorhynchus clarkii*) and rainbow trout (*O. mykiss*) are major intraguild predators of stickleback, but both occur in both the littoral and pelagic zones (James and Kelso 1995; Vamosi and Schluter 2002) and do not eliminate either niche for stickleback and so are not considered further here. Prickly sculpin (*C. asper*) are a benthic intraguild predator, and their presence selects for a more limnetic stickleback ecotype (Ingram et al. 2012). We therefore collected data on the presence/absence of prickly sculpin in all sampling locations from Hutchinson et al. (2020), Miller et al. (2019), Atkinson (2016), Dennenmoser et al. (2015), and Vamosi (2003).

The pH (Haenel et al. 2019) and dissolved calcium concentrations (Giles 1983) of lake water have previously been associated with external bony armor in stickleback (a trait which varies between benthic and limnetic ecotypes). Therefore, we also measured these variables, the former with a calibrated pH meter (Multi 340i, WTW, Weilheim, Germany) and the latter were obtained by collecting two filtered water samples (one acidified with 2% nitric acid and one frozen) from each lake. The dissolved calcium concentration (to the nearest mg/L) was then measured from the water samples at the Division of Agriculture and Environmental Science at the University of Nottingham by inductively coupled plasma mass spectrometry.

### Stickleback Sampling

Sticklebacks were caught using unbaited minnow traps set overnight from the lake shores during spring of 2015 (all stickleback ecotypes move to the shallows during the spring to breed). Samples of between 10 and 63 individuals (see supplementary table S1, Supplementary Material online for lake-specific sample sizes) were taken from each lake and transported to a rental property in aerated lake water for processing. Immediately prior to processing, fish were euthanized with an overdose of tricaine methanesulfonate (“MS222”) (400 mg/L) and killed by destruction of the brain, in accordance with Schedule One of UK Home Office regulations and with the approval of the University of British Columbia Animal Care Committee (UBC animal care certificate A11-0402). Fin clips were immediately taken and stored in 90% ethanol for later genomic analyses.

### Identification of Benthic–Limnetic Divergence

#### Phenotypic Quantification

Fish sampled from lakes containing species pairs (PAXT and PRIE) were visually classified as benthic or limnetic at the time of capture as well as being later measured for all phenotypic traits.

To determine body size, fish were blotted and weighed to the nearest milligram. To assess body shape differences, each stickleback's left side was photographed using a tripod-mounted digital SLR camera fitted with a macro lens and macro digital ring flash. Images were scaled, and 13 landmarks were placed on each image using tpsDig, version 2.16 (Rohlf 2010). Landmark data were then exported to MorphoJ, version 1.06d (Klingenberg 2011). A Procrustes fit was performed to align specimens by their main axes and remove size and rotation bias. Differences between lakes were identified using a Procrustes analysis of variance (ANOVA) with lake as the classifier. Allometric variation in body shape was removed by taking the residuals of a multivariate partial least squares regression against log centroid size, and the regression was pooled within lakes because the Procrustes ANOVA indicated differences between group centroids (Reist 1986). Regression residuals were exported into R, version 3.5.2 (R.Core.Team 2018), where they were standardized and scaled, and variation in body shape was reduced to a single axis using a PCA, implemented by singular value decomposition. This principal axis (shape PC1) was used to describe differences in body shape in all further analyses.

To assess differences in body armor, fish were first bleached and then stained with alizarin red to highlight external skeletal structures following standard procedure (Peichel et al. 2001). Fish were then rephotographed as above, images were scaled, and counts of lateral plate number, alongside measurements of standard length, first and second dorsal spine length, longest plate length, pelvis height, pelvis length, and pelvic spine length were taken (continuous elements to the nearest 0.01 mm) using ImageJ, version 1.52a (Schneider et al. 2012). All continuous armor variables (thus excluding plate number, which was independent of body size in our dataset) were size-standardized by taking the residuals of a regression against standard length. Body armor variables were highly correlated; thus, we used a PCA to reduce variation in body armor variables to a single axis: armor PC1. Armor PC1 was used to describe differences in body armor in all further analyses.

Finally, the left primary gill arch was extracted from each individual. For each gill arch, the total number of gill rakers were counted, and the mean gill raker length was calculated by taking the mean of the length of the longest three rakers on each arch, measured to the nearest micrometer.

#### Genomic SNP Analyses

DNA was isolated from fin tissue using Quiagen Blood and Tissue DNA purification kits. RAD-seq data were generated following Magalhaes et al. (2016). BAM files were produced following Magalhaes et al. (2016). Variants were called from per-individual BAM files to create a single VCF file using the Stacks pipeline (Catchen et al. 2013) in Stacks, version 1.47. The POPULATIONS program in Stacks was run with the following filters: SNPs present in <50% of individuals within a population were removed; SNPs with a minor allele frequency <0.05 were removed; and SNPs that were not present in all of the populations were removed. VCFtools, version 0.1.16 (Danecek et al. 2011), was then used to remove sites with mean depth values (over all individuals) <6 and >200, sites with >25% missing data, sites with a minor allele count over all individuals <2, and the sex chromosome (XIX). This pipeline produced an overall dataset of 12,756 SNPs for 333 individuals across the 21 lakes (dataset 1). This dataset was then subject to further filtering for some analyses, and detailed information about individual RAD datasets is given in table 1.

#### Linkage Disequilibrium

Sets of loci that have a tendency to be inherited together, and thus are highly correlated, tend to be affected by the same evolutionary processes and so contain useful information for identifying the characteristics of the processes affecting each set of linked loci, for example, whether divergence is likely linked to small genomic regions, for example, inversions, or is genome wide. To investigate whether any groups of linked loci would distinguish the genomic clusters identified in other genomic analyses, we performed an LDna using the LDna package in R. The *r*^2^ linkage disequilibrium matrix was generated using dataset 1 (12,756 SNPs) in Plink version 1.9 (Chang et al. 2015). For the extractClusters step of LDna, the minimum number of edges was set to 100 and Φ was set to 5. SNPs in each LD cluster were extracted from dataset 1 using VCFtools, VCF files were read into R using the vcfR package, and a PCA of the SNPs in each LD cluster was performed using the adegenet (Jombart 2008) package.

Many genomic tools, however, rely on the assumption that variants are independent, and therefore, SNPs in linkage disequilibrium must be removed for such analyses. To that end, we estimated linkage disequilibrium across the genome as a whole by calculating pairwise *R*^2^ values in 100 kb sliding windows using Plink2 version 2.00a2.3. *R*^2^ values range between 0 (no linkage) and 1 (complete linkage), and therefore, a relatively conservative LD threshold was set at *R*^2^ > 0.2. Thinning dataset 1 (12,756 SNPs) to unlinked loci resulted in a dataset with 9,668 retained SNPs.

#### Genomic Patterns

We used fineRADstructure (Malinsky et al. 2018) to construct a coancestry matrix using the primary SNP set including all 333 individuals (dataset 1, 12,756 SNPs). Prior filtering for linkage disequilibrium should not be performed for analyses using the RADpainter tool as it efficiently estimates the effective number of loci in mapped data files during the analysis, and this forms part of the basis of coancestry estimation (Malinsky et al. 2018). The fineSTRUCTURE (Lawson et al. 2012) clustering algorithm was run with a burn-in of 100,000 iterations followed by 100,000 sampled iterations, and the tree building algorithm was run with a burn-in of 10,000 iterations. We then performed a PCoA using a dataset filtered for linkage and filtered to remove all known QTLs in stickleback to reduce any bias caused by selection (dataset 2, 6215 SNPs, see Phylogenetic Analyses section below for details of QTL filtering). The PCoA was performed using Euclidean distances with the package adegenet in R. VCF files were converted to genpop format for input to adegenet using PGDSpider, version 2.1.1.5 (Lischer and Excoffier 2012).

#### Phenotypic Divergence

Genomic analyses grouped all fish into two broad genomic clusters, cluster 1 and cluster 2. Although marine fish were grouped with the freshwater fish in cluster 1, they were treated as a third, separate group in all further analyses. Additionally, they were excluded from most subsequent genetic analyses because their presence in freshwater/coastal areas is transient (they migrate to shallow coastal areas only in the spring to breed) and they represent the likely ancestral state of all freshwater populations (Bell and Foster 1994). To determine the phenotype of these three groups (marine, cluster 1 and cluster 2), we calculated the mean of each phenotypic variable (weight, number of gill rakers, mean raker length, armor PC1, and shape PC1) for each group. To test whether the means of each phenotypic variable in each of the three groups were significantly different from one another, linear mixed models were performed using the nlme package (Pinheiro et al. 2018), with lake included as a random effect and group (marine, cluster 1, cluster 2) as a fixed effect. For models showing a significant effect of group, post hoc pairwise comparisons were performed using estimated marginal means, implemented using the emmeans package (Lenth 2019) in R. *P* values for post hoc comparisons were adjusted for multiple testing using the false discovery rate method (Benjamini and Hochberg 1995).

#### Phylogenetic Analyses

Prior to phylogenetic analysis, we filtered our master dataset (dataset 1) to remove all known QTLs in stickleback. QTLs are loci of large effect and thus most likely to influence tree topologies in phylogenetic reconstruction. They are also extremely well mapped in stickleback (Peichel and Marques 2017), allowing for avoidance of potential biases caused by selection on these loci. This entailed removing all SNPs falling within any of the 188,257,608 bp (approximately 41% of the stickleback genome) identified in Peichel and Marques (2017) as containing known QTL in three-spined stickleback. This specifically included QTL for benthic–limnetic differences in body shape, defense (antipredator armor), feeding (trophic morphology), and pigmentation as well as QTL for many other traits that vary among stickleback populations including body size, behavior, swimming, reproduction, respiration, and sensory system differences. Data for QTL were downloaded from Peichel and Marques (2017), converted to BED format, and removed from the VCF file using VCFtools. This reduced the number of SNPs from 12,756 to 8,351. We then ensured approximate linkage equilibrium of remaining markers by removing all SNPs with an *R*^2^ value >0.2 using Plink version 1.9. This left 6,215 SNPs, dataset 2.

To construct a phylogeny of all sequenced individuals, we used a bootstrapped maximum likelihood-based approach, implemented in the hybrid version of RAxML (to allow multithreading), version 8.2.12 (Stamatakis 2014). The VCF file was converted to phylip format for input to RAxML using python version 3.8.2. RAxML was run with a GTR-GAMMA model of substitution rate heterogeneity, with an automatic bootstrap replicate halting using the autoMRE function, and with the default settings for all other parameters. We determined the optimal nucleotide substitution model (GTRGAMMA) using MrModeltest version 2.3 (Nylander 2004) in PAUP* version 4.0 (Swofford 2002).

To assess the robustness of the maximum likelihood phylogeny, we also performed topology weighting using TWISST (Martin and Van Belleghem 2017). Topology weighting was carried out on four freshwater populations, with a single marine sequence (from LICA) as the outgroup. The freshwater populations were selected to contain two pairs of geographically proximal populations, with one pair from either side of the Georgia strait (NORT and BEAV, and DOUG and KLEN) and with one population from each pair falling in cluster 1 and the other in cluster 2. The linkage and QTL filtered dataset was filtered to contain all individuals from each of the four freshwater populations and a single individual from LICA (TWISST only accepts a single sequence as an outgroup), using VCFtools (dataset 3, 6,215 SNPs). The VCF file was converted to .geno format, and maximum likelihood trees were estimated in phyml (Guindon et al. 2010) in sliding windows of 50 bp using Python 2.7.15 and the scripts available with TWISST. Topology weightings were then computed using Python 3.8.2, and topologies were visualized in R.

#### Phylogenetic Signal in Phenotypic Traits

To estimate phylogenetic signal, the phylogeny constructed in RAxML was imported into R using the ape package (Paradis and Schliep 2019); individual nodes were collapsed to leave a single node per population, with the exception of the two marine populations, which were both collapsed into one node using the phytools (Revell 2012) and phangorn (Schliep 2011) R packages. Phenotypic trait data (weight, number of gill rakers, mean raker length, armor PC1, and shape PC1) were added to the tree tips, and phylogenetic signal and associated *P* values for each trait were estimated using the package phylosignal (Keck et al. 2016). We used Pagel's λ (Pagel 1999) to estimate phylogenetic signal as this statistic performs well compared with others available and has a low type 1 error rate (Freckleton et al. 2002; Muenkemueller et al. 2012; Molina-Venegas and Rodriguez 2017). *P* values are calculated using likelihood ratio tests that compare the observed λ statistic with a phylogenetically independent trait distribution. We also simulated data for five additional traits to be distributed randomly with regard to phylogeny. Simulated traits were tested alongside the real phenotypic variables for comparison.

As we aim to detect whether benthic and limnetic characteristics have evolved a single time or repeatedly across the radiation, we also used the R package SURFACE (Ingram and Mahler 2013) to estimate the most likely number of different selection regimes (*k*) and instances of convergent evolution (*c*) by identifying the best fitting model of trait evolution for our phylogeny and associated phenotypic traits. SURFACE begins by fitting a single peak Ornstein–Uhlenbeck (OU) model (which allows for a single adaptive optimum and variation in the parameter α, which describes the strength of selection toward that optimum) by maximum likelihood. It then sequentially adds adaptive peaks to the model in a step-wise process and accepts each more complex model until Akaike information criterion (AIC) values are no longer improved. SURFACE then attempts to collapse regimes with the same optima in a process of step-wise backwards selection whereby if multiple optima are the same, the AIC of the model is improved by reducing the number of model parameters. We ran SURFACE using the same collapsed phylogeny and associated trait data that were generated to estimate phylogenetic signal. The tree was converted to the ouchtree format, and the best fitting model of trait evolution was estimated under an AIC threshold of 0 (any improvement in AIC should be accepted) using the SURFACE R package.

#### Relationship between Phenotype and Environment

To investigate associations between environmental characteristics and divergence in phenotypic traits, we used a PGLS approach, so that phylogenetic signal could be accounted for in the models, using the ape (Paradis and Schliep 2019), nlme (Pinheiro et al. 2018), and geiger (Harmon et al. 2008) packages in R. Marine fish were excluded from all phenotype–environment modeling because our main aim was to detect effects in relation to the freshwater benthic and limnetic phenotypes in BC, and although marine fish have a limnetic phenotype, we found them to differ phenotypically from the freshwater limnetic fish in BC. Separate models were run for each phenotypic trait (weight, number of gill rakers, mean gill raker length, armor PC1, and shape PC1). Models were fitted by maximum likelihood, and we began with all environmental variables in each model (mean lake depth [m], lake area [km^2^], presence/absence of prickly sculpin, pH, and calcium concentration [mg/L]). Terms were then removed sequentially, with the least significant terms removed first, until the reduced model was no longer a significant improvement on the fuller model under the *P* < 0.05 threshold. Model comparison was conducted using Wald tests. Phylogenetic effects for each phenotypic trait were accounted for in each model following the principles set out in Mazel et al. (2016). We first transformed the phylogeny for each phenotypic trait under a lambda model with lambda specified as the lambda estimate for that phenotypic trait in the phylogenetic signal analyses. The phylogenetic variance–covariance matrices were computed from the transformed trees using the ape package and converted to correlation matrices, which were used to specify phylogenetic correlation of errors in the models.

## Supplementary Material

msad191_Supplementary_Data

## Data Availability

BAM files of the aligned reads for each individual and corresponding sample information have been deposited in the European Nucleotide Archive database under the project PRJEB65359, with the sample accession numbers ERS16289139 and ERS16289471. Phenotypic and environmental data have been deposited in the University of Nottingham's digital data repository under the doi: 10.17639/nott.7328. ***Conflict of interest statement.*** The authors declare no competing interests.
