# Supplementary material for: On the Origins of Phenotypic Parallelism in Benthic and Limnetic Stickleback
Source: Mol Biol Evol. 2023 Aug 31;40(9):msad191. doi: 10.1093/molbev/msad191 (PMC10490448; doi:10.1093/molbev/msad191)
Supplement: msad191_Supplementary_Data [file msad191_supplementary_data.zip › BC supplementary material MBE.docx]

Supplementary material

for

**On the origins of phenotypic parallelism in benthic and limnetic stickleback**

Laura L. Dean^1^*, Isabel Santos Magalhaes^1,2^, Daniele D’Agostino^1,3^ Paul Hohenlohe^4^ and Andrew D. C. MacColl^1^

^1^School of Life Sciences, The University of Nottingham, University Park, Nottingham, NG7 2RD, UK
^2^School of Health and Life Sciences, Whitelands College, University of Roehampton, London SW15 4JD, UK
^3^Water Research Center, New York University Abu Dhabi, PO Box 129188, Abu Dhabi, United Arab Emirates

^4^Institute for Bioinformatics and Evolutionary Studies, Department of Biological Sciences, University of Idaho, Moscow, ID, USA.

Correspondence: [lldean18@gmail.com](mailto:lldean18@gmail.com)

Correspondence: andrew.maccoll@nottingham.ac.uk

**Supplementary Tables**

**Table S1**

**Description of sample sites.** Sample sizes are shown for phenotypic (n pheno) and RAD (n RAD) analyses. Sal refers to salinity classifications, Fr: freshwater (absolute conductivity <500 μS/cm), Br: brackish (absolute conductivity 20,000-35,000 μS/cm). Sampling locations are given by latitude followed by longitude.

| **Lake ID** | **Lake name** | **n pheno** | **n RAD** | **Genomic cluster** | **Mean depth (m)** | **Area (km^2^)** | **Sculpin** | **Sal** | **Location** |
| --- | --- | --- | --- | --- | --- | --- | --- | --- | --- |
| AMBR | Ambrose lake | 29 | 17 | 1 | 13.3 | 0.2980 | Yes | Fr | 49°44'3"N; 124°1'16"W |
| BEAV | Beaver lake | 30 | 19 | 1 | 3.5 | 0.1932 | Yes | Fr | 48°48'42"N; 124° 4'51"W |
| BRAN | Brannen lake | 30 | 19 | 2 | 11.6 | 1.0866 | Yes | Fr | 49°12'55"N; 124°3'18"W |
| BULL | Bullocks lake | 30 | 17 | 2 | 4.0 | 0.0940 | No | Fr | 48°52'25N; 123°30'33"W |
| CRAN | Cranby lake | 30 | 20 | 2 | 3.2 | 0.4460 | No | Fr | 49°41'36"N; 124°30'42"W |
| DOUG | Dougan lake | 30 | 16 | 2 | 8.5 | 0.1000 | No | Fr | 48°42'53"N; 123°36'48"W |
| GARD | Garden bay | 10 | 9 | 1 | 10.0 | 0.6236 | Yes | Fr | 49°39'1"N; 124°1'23"W |
| HOGG | Hoggan lake | 30 | 20 | 2 | 3.0 | 0.1970 | No | Fr | 49°9'7"N; 123°49'43"W |
| HOTE | Hotel | 35 | 17 | 1 | 5.9 | 0.2522 | No | Fr | 49°38'26"N; 124°3'3"W |
| KENN | Kennedy lake | 30 | 19 | 1 | 38.0 | 65.0000 | Yes | Fr | 49°3'59"N; 125°28'2"W |
| KIRK | Kirk lake | 30 | 18 | 2 | 8.3 | 0.0830 | No | Fr | 49°44'22"N; 124°34'57"W |
| KLEN | Klein lake | 30 | 17 | 2 | 12.0 | 0.1350 | No | Fr | 49°43'53"N; 123°58'23"W |
| LICA | Little Campbell river | 34 | 10 | 1 | 3.0 | 74.400 | Yes | Br | 49°0'51"N; 122°45'32"W |
| LILY | Lily lake | 30 | 19 | 1 | 2.2 | 0.1214 | Yes | Fr | 49°36'44"N; 124° 1'17"W |
| NORT | North lake | 14 | 14 | 1 | 10.1 | 0.1279 | Yes | Fr | 49°44'60"N; 123°58'27"W |
| OYST | Oyster lagoon | 30 | 10 | 1 | 1.3 | 0.0200 | Yes | Br | 49°36'49"N; 124°1'47"W |
| PAXT | Paxton lake | 63 | 9 | 1 / 2 | 6.2 | 0.1700 | No | Fr | 49°42'22"N; 124°31'24"W |
| PRIE | Priest lake | 57 | 9 | 1 / 2 | 5.4 | 0.4434 | No | Fr | 49°44'50"N; 124°33'52"W |
| SPRO | Sproat lake | 30 | 19 | 1 | 65.5 | 37.7500 | Yes | Fr | 49°16'0"N; 125°2'13"W |
| STOW | Stowell lake | 30 | 17 | 2 | 4.6 | 0.0564 | No | Fr | 48°46'54"N; 123°26'37"W |
| TROU | Trout lake | 30 | 18 | 2 | 5.8 | 0.0756 | No | Fr | 49°30'26"N; 123°52'37"W |

**Table S2**

**SNPs in linkage cluster 10 that lie within genes.** Genome location of SNPs falling within coding regions and the associated gene and gene ID for each SNP.

| **Chromosome** | **SNP** | **Gene name** | **Ensembl gene ID** |
| --- | --- | --- | --- |
| groupI | 138067 |  | ENSGACG00000004458 |
| groupI | 1913469 |  | ENSGACG00000005744 |
| groupI | 14210802 |  | ENSGACG00000011908 |
| groupI | 22123269 |  | ENSGACG00000014464 |
| groupII | 20248077 | NELL1 | ENSGACG00000017098 |
| groupIV | 4186906 | pde6a | ENSGACG00000016811 |
| groupV | 291741 | kat6b | ENSGACG00000002173 |
| groupV | 1957242 | glud1b | ENSGACG00000002813 |
| groupV | 2216160 | cyth1b | ENSGACG00000002991 |
| groupVI | 2259976 | cpeb3 | ENSGACG00000002944 |
| groupVI | 6422836 | fbxolla | ENSGACG00000005432 |
| groupVI | 13171573 | sdccag8 | ENSGACG00000010549 |
| groupVII | 20193529 | dclk1a | ENSGACG00000020560 |
| groupX | 8795111 | fam126a | ENSGACG00000006077 |
| groupXIII | 9178337 | ube3b | ENSGACG00000009166 |
| groupXIII | 15603103 | arvcfb | ENSGACG00000013003 |
| groupXIII | 17477236 | krt1-c5 | ENSGACG00000013907 |
| groupXIV | 4013936 | dnajc21 | ENSGACG00000016480 |
| groupXV | 9968890 | ppp2r3a | ENSGACG00000010930 |
| groupXV | 14295031 | odc1 | ENSGACG00000012974 |
| groupXVI | 119418 |  | ENSGACG00000001436 |
| groupXVI | 210791 | pde9a | ENSGACG00000001450 |
| groupXVII | 840001 | ccdc51 | ENSGACG00000003571 |
| groupXVII | 6327443 | trim62.1 | ENSGACG00000007462 |
| groupXXI | 2692493 | nrros | ENSGACG00000002094 |
| scaffold_114 | 90942 | itpr3 | ENSGACG00000000253 |
| scaffold_114 | 90942 |  | ENSGACG00000000233 |
| scaffold_27 | 3558934 | wdr82 | ENSGACG00000001254 |

**Table S3**

**Linear mixed model results for phenotypic differences between groups.** Table shows the results of linear mixed models testing for differences between three groups (marine, genomic cluster 1 and genomic cluster 2) in five phenotypic traits, with population as a random effect.

| **Trait** | ***F*** | ***df*** | ***p*-value** |
| --- | --- | --- | --- |
| Weight | 4.4219 | 2 | 0.0257 |
| N gill rakers | 5.4898 | 2 | 0.0126 |
| Raker length | 12.4074 | 2 | 0.0003 |
| Armour PC1 | 8.1948 | 2 | 0.0025 |
| Shape PC1 | 23.0643 | 2 | <0.0001 |

*F* describes the likelihood ratio test statistic, *df* describes degrees of freedom. *P*-values < 0.05 are highlighted in bold.

**Table S4**

**Post-hoc Estimated marginal means test results for pairwise phenotypic differences between groups.** Table shows the results of post-hoc estimated marginal means tests to determine pairwise differences between groups (marine, genomic cluster 1 and genomic cluster 2) when phenotypes differed significantly between groups in linear mixed models.

| **Trait** | **Groups compared** | **Estimate** | **SE** | ***df*** | **Adjusted *p*-value** |
| --- | --- | --- | --- | --- | --- |
| Weight | limnetic-like – benthic-like | 0.2453 | 0.2400 | 20 | 0.3188 |
|  | marine – benthic-like | -1.0119 | 0.4193 | 20 | **0.0383** |
|  | marine – limnetic-like | -1.2572 | 0.4232 | 20 | **0.0227** |
| N gill rakers | limnetic-like – benthic-like | -2.0254 | 0.6387 | 20 | **0.0144** |
|  | marine – benthic-like | -1.9978 | 1.1211 | 20 | 0.1349 |
|  | marine – limnetic-like | 0.0276 | 1.1304 | 20 | 0.9808 |
| Raker length | limnetic-like – benthic-like | -0.1799 | 0.0639 | 20 | **0.0107** |
|  | marine – benthic-like | -0.5286 | 0.1121 | 20 | **0.0004** |
|  | marine – limnetic-like | -0.3487 | 0.1130 | 20 | **0.0088** |
| Armour PC1 | limnetic-like – benthic-like | -2.2551 | 0.6914 | 20 | **0.0069** |
|  | marine – benthic-like | -3.8791 | 1.2155 | 20 | **0.0069** |
|  | marine – limnetic-like | -1.6240 | 1.2250 | 20 | 0.1999 |
| Shape PC1 | limnetic-like – benthic-like | -2.4654 | 0.4697 | 20 | **<0.0001** |
|  | marine – benthic-like | -4.5652 | 0.8202 | 20 | **<0.0001** |
|  | marine – limnetic-like | -2.0998 | 0.8280 | 20 | **0.0197** |

Estimates describe the mean difference between groups, SE describes the standard error of the estimates and *df* describe degrees of freedom. *P*-values are adjusted for multiple testing using the FDR method. *P*-values < 0.05 are highlighted in bold.

**Table S5**

**Effects of the environment on the distribution of benthic-limnetic phenotypic traits.** Table shows the results of phylogenetic generalised least squares analyses (PGLS) on each phenotypic trait. *P*-values < 0.05 are highlighted in bold. *P*-values were adjusted for multiple testing using the FDR method.

| **Phenotypic trait** | **Environmental variable** | ***df*** | **Wald statistic** | ***p*-value** | **Adjusted *p*-value** |
| --- | --- | --- | --- | --- | --- |
| Armour PC1 | Lake area | 1 | 1.5877 | 0.2077 | 0.5077 |
|  | Ca concentration | 1 | 3.2274 | 0.0724 | 0.2172 |
|  | Mean lake depth | 1 | 0.0883 | 0.7663 | 0.8969 |
|  | pH | 1 | 1.6609 | 0.1975 | 0.3950 |
|  | Sculpin presence | 1 | 17.6411 | **0.0000** | **0.0002** |
|  |  |  |  |  |  |
| N rakers | Lake area | 1 | 0.0461 | 0.8300 | 0.8300 |
|  | Ca concentration | 1 | 0.3525 | 0.5527 | 0.6088 |
|  | Mean lake depth | 1 | 0.0327 | 0.8564 | 0.8969 |
|  | pH | 1 | 0.1860 | 0.6663 | 0.7882 |
|  | Sculpin presence | 1 | 0.7284 | 0.3934 | 0.4721 |
|  |  |  |  |  |  |
| Raker length | Lake area | 1 | 6.8857 | **0.0087** | 0.0521 |
|  | Ca concentration | 1 | 1.4304 | 0.2317 | 0.4634 |
|  | Mean lake depth | 1 | 0.0168 | 0.8969 | 0.8969 |
|  | pH | 1 | 0.5091 | 0.4755 | 0.7133 |
|  | Sculpin presence | 1 | 2.6932 | 0.1008 | 0.1512 |
|  |  |  |  |  |  |
| Shape PC1 | Lake area | 1 | 0.0641 | 0.8001 | 0.8300 |
|  | Ca concentration | 1 | 0.2620 | 0.6088 | 0.6088 |
|  | Mean lake depth | 1 | 0.1346 | 0.7137 | 0.8969 |
|  | pH | 1 | 1.7163 | 0.1902 | 0.3950 |
|  | Sculpin presence | 1 | 3.8490 | **0.0498** | 0.0996 |
|  |  |  |  |  |  |
| Weight | Lake area | 1 | 0.1549 | 0.6939 | 0.8300 |
|  | Ca concentration | 1 | 0.5770 | 0.4475 | 0.6088 |
|  | Mean lake depth | 1 | 16.8205 | **0.0002** | **0.0013** |
|  | pH | 1 | 7.9713 | **0.0048** | **0.0285** |
|  | Sculpin presence | 1 | 0.0013 | 0.9714 | 0.9714 |

**Supplementary Figures**

**
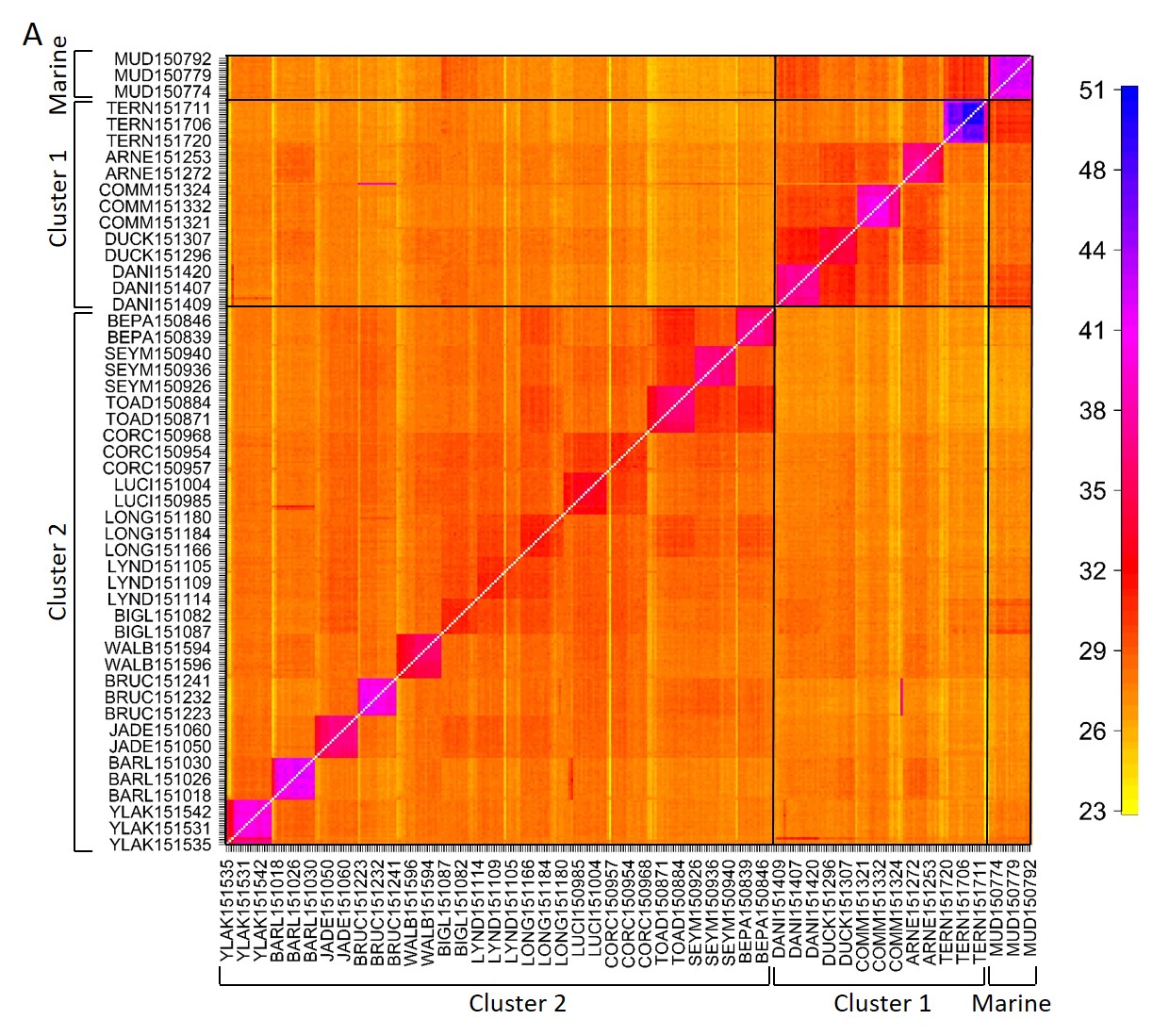
**

**
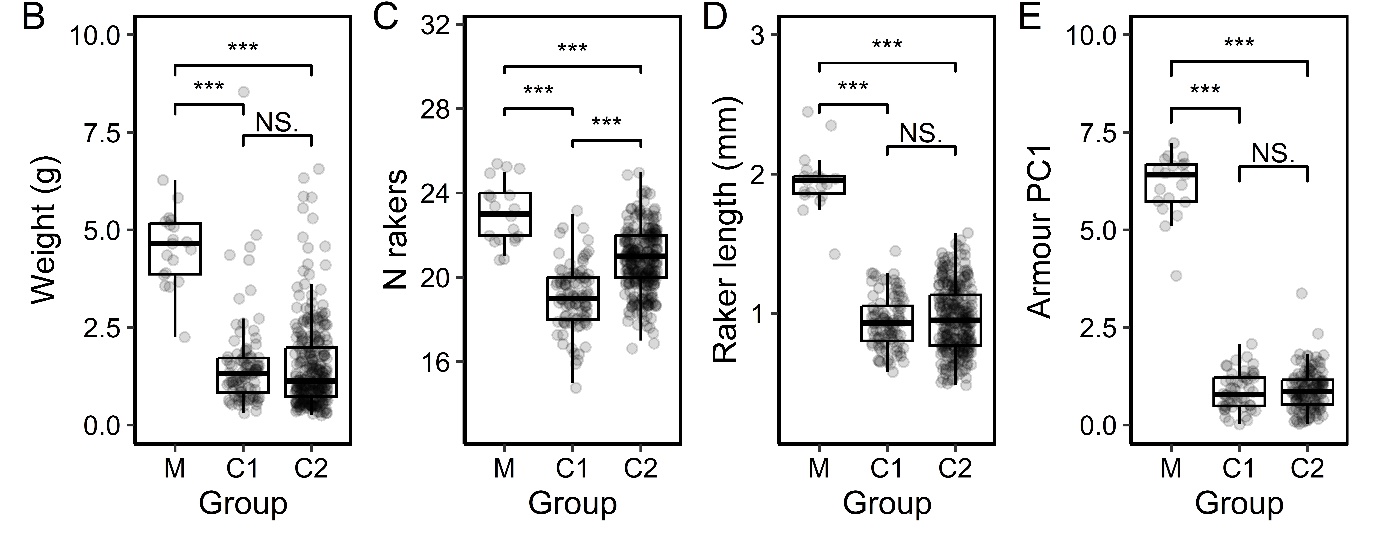
**

**Figure S1.** A. Co-ancestry matrix of 49 individuals from 18 freshwater stickleback populations and one marine population in Alaska. Black lines separate marine fish and the broader genetic clusters. Analysis was based on 14,882 SNPs from data collected and processed in an identical manner to that in this study see supplementary methods and Magalhaes et al. [1] for detailed methods and population information. B – E Phenotypic differences between marine fish and two freshwater genetic clusters in Alaska. Circles represent individuals. Abbreviations: M – marine, C1 – cluster 1, C2 – cluster 2. Brackets and asterisks indicate significance thresholds of post-hoc estimated marginal means tests between groups: NS. indicating *p* > 0.05, ** indicating *p* < 0.01 and *** indicating *p* < 0.001. All *p* values were adjusted for multiple comparisons.


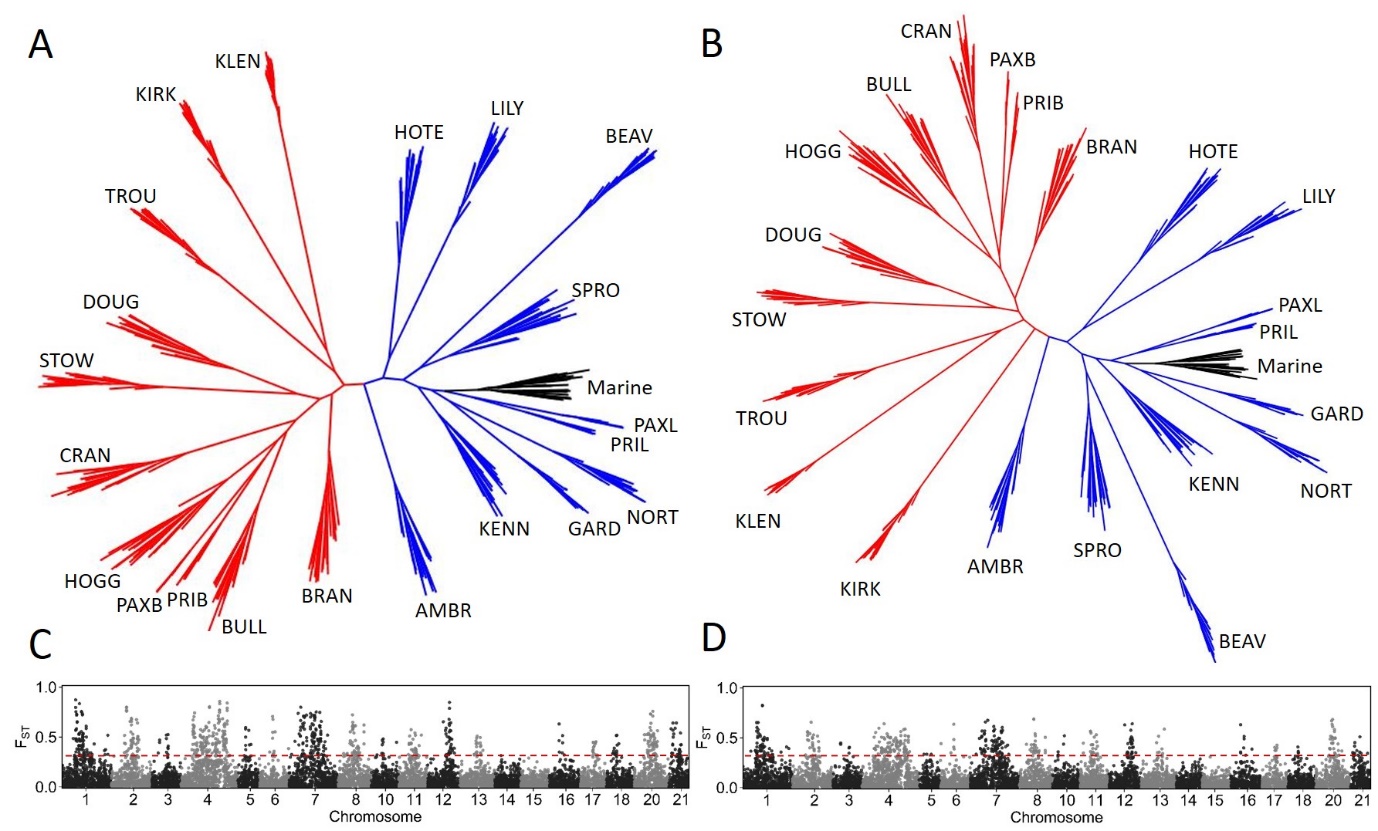


**Figure S2**. Maximum likelihood phylogenies of 333 BC stickleback, constructed using SNPs filtered to exclude (A) all 537 SNPs with Fst between cluster 1 and cluster 2 >= 0.35, leaving 12,219 SNPs and (B) all 367 SNPs with Fst between sculpin present and sculpin absent populations >= 0.35, leaving 12,389 SNPs. Black indicates marine; blue, cluster 1 and red, cluster 2. Per-site Fst plots for cluster 1 vs. cluster 2 and sculpin presence vs. absence are shown in (C) and (D) respectively. Dashed red lines indicate the Fst cut off of 0.35 used to filter SNP sets prior to phylogenetic reconstruction in (A) and (B).


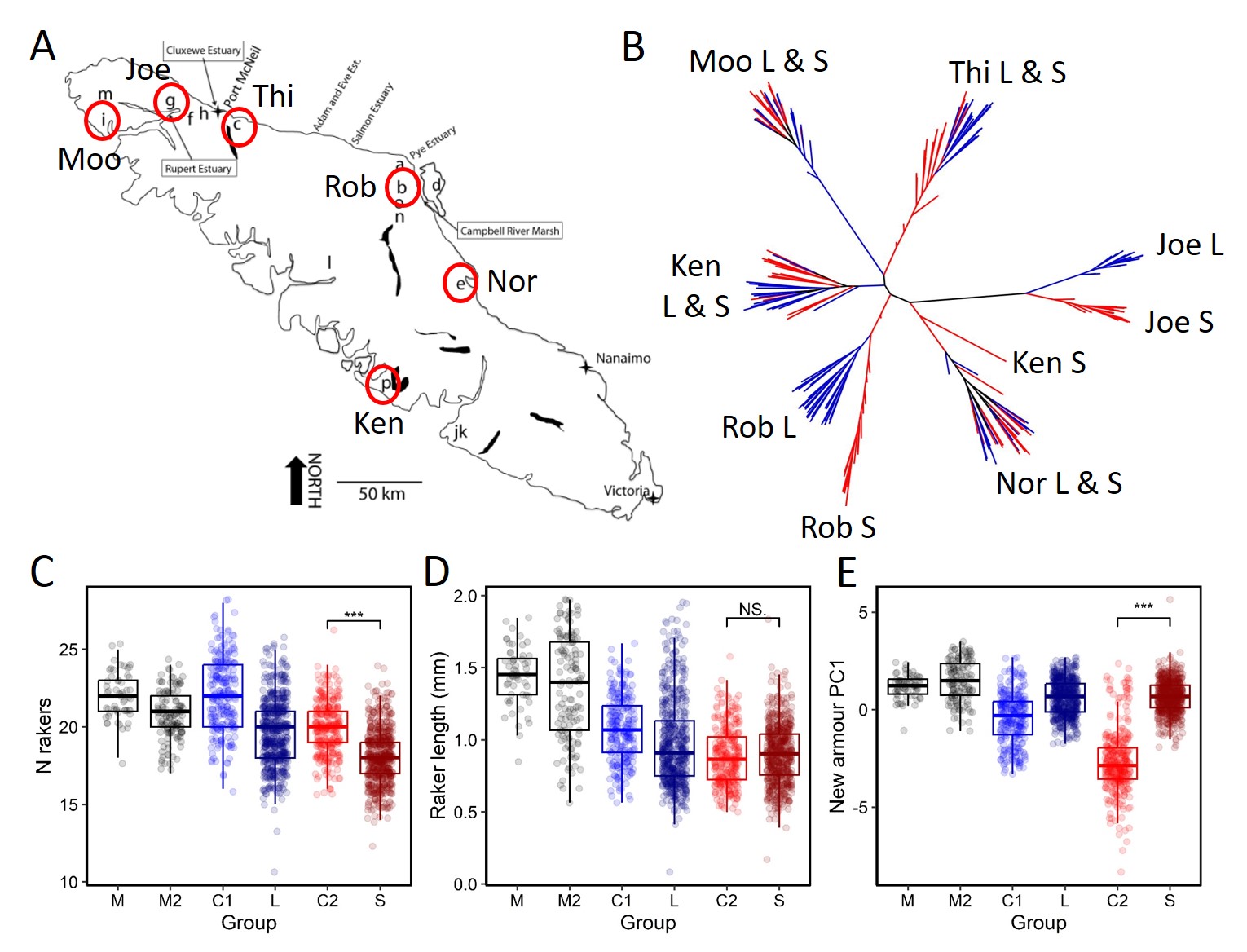


**Figure S3.** Analysis of data from Stuart et al. [2]. (A) Map of Vancouver island, modified from Stuart et al. [2], highlighting the locations of the six lake – stream pairs selected for comparative phylogenetic reconstruction. (B) Maximum likelihood phylogeny of 201 individuals from the 6 lake – stream population pairs shown in (A). Lake individuals are shown in blue and stream in red, abbreviated population names are given followed by L for lake individuals and S for stream individuals. (C-E) Comparison of phenotypic traits between marine (M), cluster 1 (C1) and cluster 2 (C2) individuals from this manuscript and marine (M2), lake (L) and stream (S) individuals sampled by Stuart et al. [2]. Circles represent individuals, brackets and asterisks indicate significance of t-tests performed to compare phenotypes between cluster 2 and stream fish. NS. indicates *p* > 0.05, and *** indicates *p* < 0.001. All *p* values were adjusted for multiple comparisons.

**
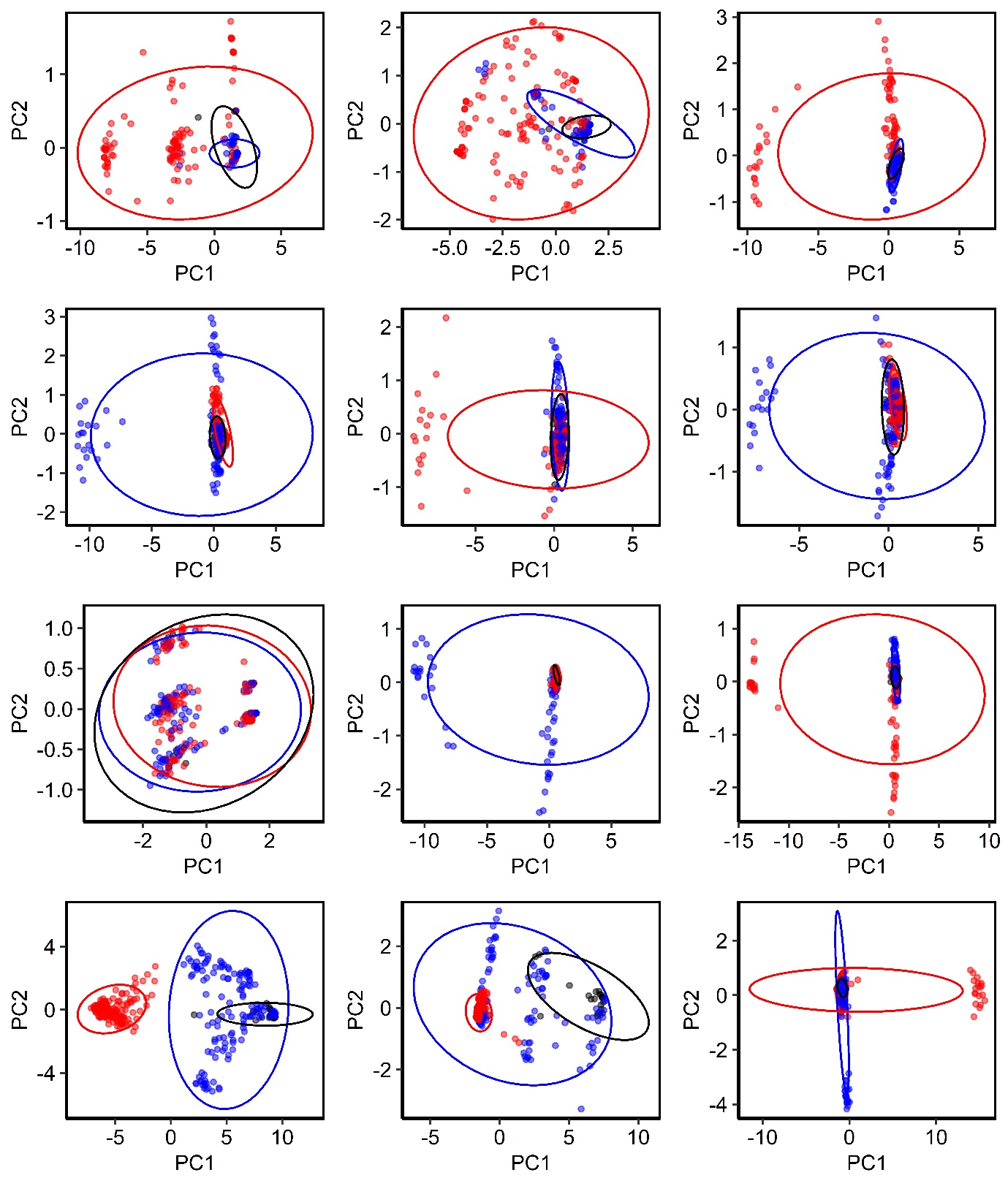
**

**Figure S4.** Principal component analyses (PCA) of each LD cluster in the LD network identified by LDna. Black circles represent marine individuals, blue circles, freshwater cluster 1 and red circles freshwater cluster 2. 95% confidence ellipses are shown for each PCA in the corresponding colours.


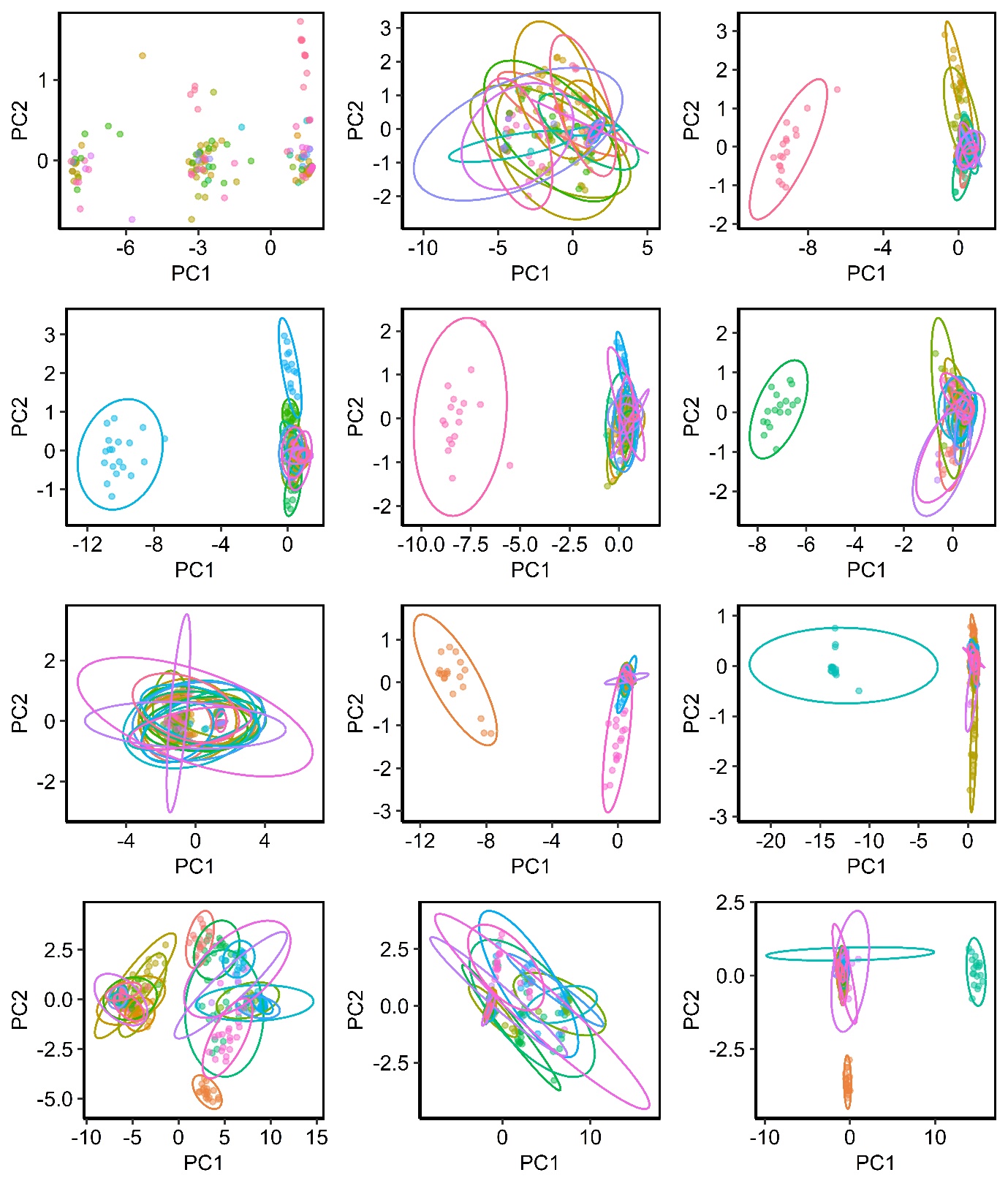


**Figure S5.** Principal component analyses (PCA) of each LD cluster in the LD network identified by LDna. Coloured circles and ellipses represent each of the 23 sampled populations.


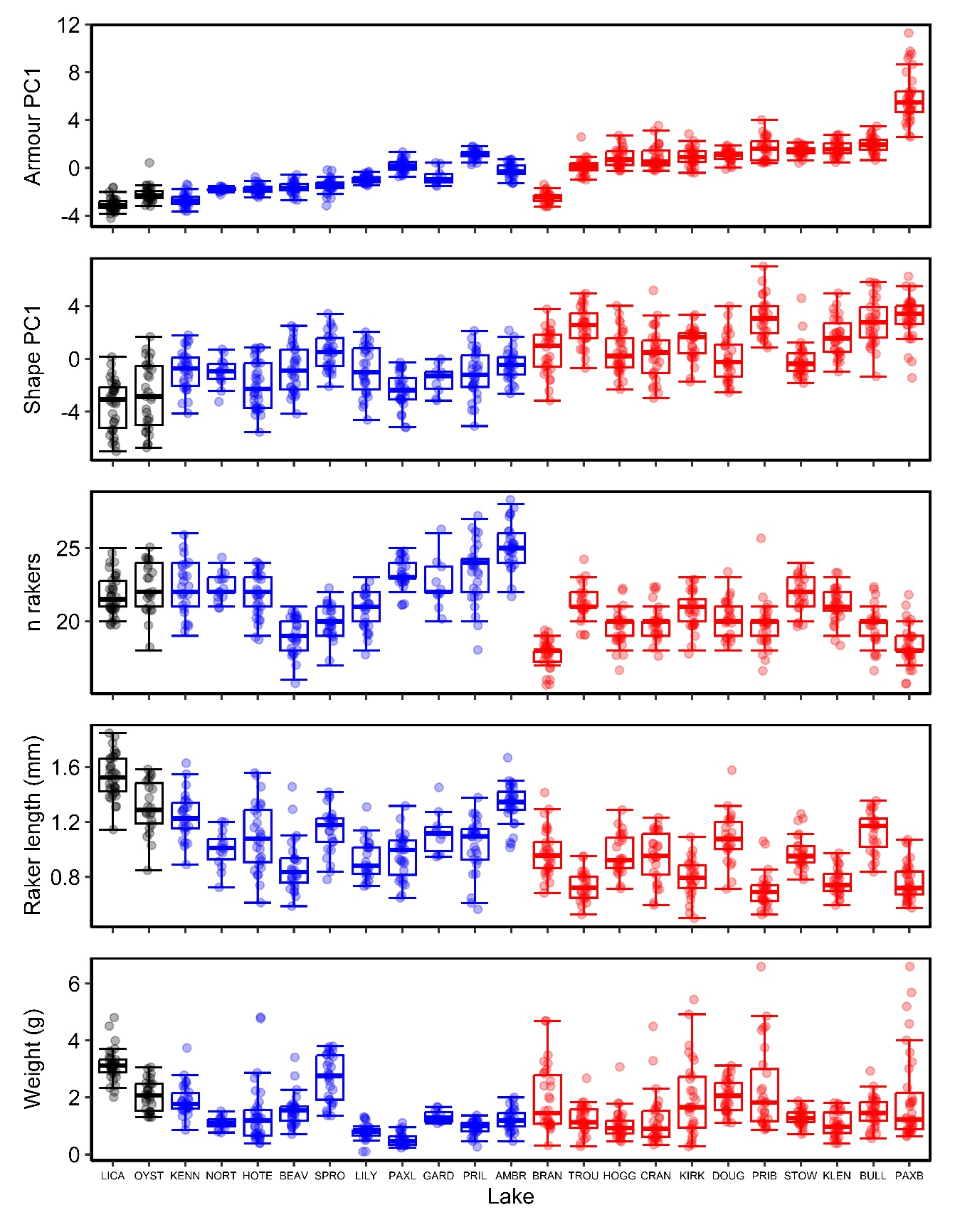


**Figure S6.** Phenotypic differences between populations. Circles represent individuals. Colours show black: marine populations, blue: cluster 1 populations, red: cluster 2 populations.

**Supplementary Methods**

Supplementary analysis of Alaskan stickleback

Genomic DNA for Alaskan stickleback was isolated and sequenced in an identical manner to that of the BC stickleback (following Magalhaes et al. [1]). Variants from Alaskan sequence data were called from per-individual BAM files in Stacks following the same methodology as for BC stickleback (SNPs with a minimum depth of coverage < 3 were removed; SNPs present in < 50% of individuals within a population were removed; SNPs with a minor allele frequency < 0.05 were removed; and SNPs that were not present in all of the populations were removed. VCFtools was then used to remove sites with mean depth values over all individuals < 6 and > 200, sites with > 25% missing data, sites with a minor allele count over all individuals < 2 and the sex chromosomes.). This pipeline resulted in an overall dataset of 14,882 SNPs for 337 individuals across 18 freshwater lakes and one marine site in Alaska. A co-ancestry matrix was constructed for Alaska using this set of 14,882 SNPs in fineRADstructure as for BC populations, but using a subset of 2-3 individuals per population. See the supplementary material in Magalhaes et al. [1] for details about Alaskan populations and for sample sizes. Population codes for populations included in this analysis were: MUD, TERN, ARNE, COMM, DUCK, DANI, BEPA, SEYM, TOAD, CORC, LUCI, LONG, LYND, BIGL, WALB, BRUC, JADE, BARL and YLAK.

Similar to in BC, the Alaskan co-ancestry plot identified two broad genomic clusters. However, in Alaska, genomic clusters represented broad geographical patterns. The first genomic cluster included marine fish and all freshwater populations from the Kenai Peninsula. The second included freshwater populations from the Mat-su valley.

Phenotypic variables (Weight, number of gill rakers, mean gill raker length, number of lateral plates, standard length, first and second dorsal spine length, longest plate length, pelvis height, pelvis length and pelvic spine length) for Alaskan stickleback populations were collected and processed in an identical manner to BC. All continuous armour variables (thus excluding plate number, which was again independent of body size in the Alaska data set) were size-standardized by taking the residuals of a regression against standard length. Principal components analysis (PCA) was used to reduce variation in body armour variables to a single axis: armour PC1.

We calculated the mean of each phenotypic variable (weight, number of gill rakers, mean raker length and armour PC1) for each genomic cluster identified in co-ancestry analyses, separating Alaskan marine fish from cluster 1 as in BC analyses. Body shape data was not available for Alaskan fish so we did not assess differences in body shape for Alaska. We compared the means of phenotypic variables across groups as for BC, but found no difference between the two Alaskan freshwater clusters in weight, length of gill rakers or armour PC1. Cluster 2 in Alaska (sharing the least common ancestry with marine fish) did have significantly more gill rakers than cluster 1.

Supplementary alternative SNP filtering and phylogenetic reconstruction

As an alternative to removing all known QTL from our master dataset prior to phylogenetic reconstruction, we also constructed phylogenies based on two further SNP sets, one excluding SNPs with a cluster 1 cluster 2 Fst >= 0.35 and one excluding SNPs with sculpin (a major selective agent for stickleback) presence – absence Fst >= 0.35. Fst was calculated for each pair of comparisons on a per-site basis using VCFtools version 0.1.17. Fst outliers (>= 0.35) were written to bed files using R version 4.2.2 and excluded from dataset 1 using VCFtools. There were 537 SNPs with Fst >= 0.35 in the cluster 1 – cluster 2 comparison, and 367 SNPs with Fst >= 0.35 in the sculpin presence – absence comparison so removing these left sets of 12,219 and 12,389 SNPs respectively for phylogenetic reconstruction. Phylogenies using these two SNP sets were constructed using RAxML following the same method as for dataset 2 in the main manuscript.

Supplementary analysis of data from Stuart et al. [2]

*Genomic analysis*

Raw fastq files generated by ddRADseq were acquired from Stuart et al. [2] from the authors with permission. Processing of genomic data was carried out in, as far as possible, an identical manner to the processing of the RADseq data in this manuscript. Fastq files for each individual from multiple sequencing runs were concatenated using a custom bash script and demultiplexed using the process_radtags feature in Stacks version 2.41, with the following settings: -i gzfastq --paired --rescue --clean --quality --inline-index --disable-rad-check --renz-1 nlaIII --renz-2 mluCI. Fastq files were unzipped and file headers modified to ensure individuals were correctly named within files. Reads for all individuals from a subset of populations sampled in Stuart et al. [2] (201 total individuals from 12 populations, 6 lake – stream pairs, selected to include watersheds across Vancouver island, see Figure S3 for sample locations) were mapped to version 82 of the Broads S1 *G. aculeatus* reference assembly using BWA version 9.3.0. BAM files were sorted and indexed using Samtools version 1.10 and SNPs were called using the ref_map.pl feature in Stacks. Sites were then filtered using the populations feature in Stacks with the following filters: to be retained a site must be present in all 12 populations, be present in at least 50% of individuals in each population have a minor allele frequency > 0.05. VCFtools, version 0.1.16, was then used to remove sites with mean depth values < 6 and > 200, sites with > 25% missing data, sites with a minor allele count over all individuals < 2, the sex chromosome (XIX) and all known QTL in *G. aculeatus* [3]. Linkage disequlibrium was then calculated in 100kb sliding windows using Plink2 version 2.00a2.3 and all sites with R^2^ >0.2 were removed. This resulted in a dataset of 3857 SNPs.

It was not possible to combine the genomic data collected by Stuart et al. [2] with that collected in this manuscript because Stuart et al. [2] used different restriction enzymes to the one used in this manuscript and so there were almost no shared sites across the two genomic datasets. However, the filtered SNP set generated from Stuart et al. [2]’s data was used to construct an independent maximum likelihood phylogeny in RAxML version 8.2.12. The aim of this analysis was to determine whether divergent selection for lake vs. stream habitats would be sufficient in the filtered dataset to produce a phylogeny in which lake individuals were monophyletic and stream individuals were monophyletic, rather than clustering by watershed. The VCF file was converted to phylip format for input to RAxML using python version 3.8.2. RAxML was run with a GTR-GAMMA model of substitution rate heterogeneity, automatic bootstrap replicate halting using the autoMRE function and with the default settings for all other parameters, as for the main phylogeny in our manuscript (Figure 4A).

*Phenotypic analysis*

Phenotypic data from all individuals sampled in Stuart et al. [2] was downloaded from the online data repository and analysed alongside data from this manuscript in R version 4.3.0. In our study we investigated five phenotypic traits, four of which (number of gill rakers, gill raker length, armour PC1 and shape PC1) were important for differentiating the two genomic clusters we identified. Stuart et al. [2] collected gill raker counts and measurements of mean gill raker length in the same standardised way as data were collected for this manuscript and so these data were directly comparable. Not all body armour variables that were used for PCA in this manuscript were collected by Stuart et al. [2], so to make the armour data comparable, we merged our phenotypic data with that of Stuart et al. [2], and regressed each of the four continuous armour measurements that were collected in both datasets (lengths of the first and second dorsal spines, length of the pelvic spine and length of the pelvis) against standard length for all individuals from both datasets combined. We then performed principal components analysis (PCA) on the regression residuals for the four continuous traits plus lateral plate number (which was independent of body size). Principal component 1 (new armour PC1) explained 72.5% of variation in these five armour traits and described increasing size of all armour variables in a very similar manner to armour PC1 in the main analysis of this manuscript. Although body shape data was also collected by Stuart et al. [2], we decided not to attempt to analyse it alongside the shape data collected in this manuscript because geometric morphometric analysis is so sensitive to the exact positioning of each landmark that we felt it would be very unlikely that our two data sets would be directly comparable. We were thus able to directly compare three phenotypic traits (gill raker number, gill raker length and new armour PC1) between individuals sampled in this manuscript and all marine, lake and stream individuals sampled by Stuart et al. [2]. We were not able to separate lake populations from Stuart et al. [2] into cluster 1 or 2 because RADseq data collected by Stuart et al. [2] used different restriction enzymes to the one used in this manuscript and so genomic data could not be combined as there were almost no shared sites across genomic datasets. We were, however, interested in whether the stream populations sampled in Stuart et al. [2] were phenotypically different from the benthic-like fish that made up genomic cluster 2 in our main analyses. We therefore performed t-tests to compare trait means for cluster 2 individuals from our analysis with stream fish from for each of the three traits compared. *P* values for t-tests were corrected for multiple testing using the FDR method.

REFERENCES

1. Magalhaes, I.S., Whiting, J.R., D'Agostino, D., Hohenlohe, P.A., Mahmud, M., Bell, M.A., Skulason, S., and MacColl, A.D.C. (2021). Intercontinental genomic parallelism in multiple three-spined stickleback adaptive radiations. Nature Ecology & Evolution *5*, 251-261.

2. Stuart, Y.E., Veen, T., Weber, J.N., Hanson, D., Ravinet, M., Lohman, B.K., Thompson, C.J., Tasneem, T., Doggett, A., Izen, R., et al. (2017). Contrasting effects of environment and genetics generate a continuum of parallel evolution. Nature Ecology & Evolution *1*.

3. Peichel, C.L., and Marques, D.A. (2017). The genetic and molecular architecture of phenotypic diversity in sticklebacks. Philosophical transactions of the Royal Society of London. Series B, Biological sciences *372*, 20150486.
